# MicroRNA-608 Promotes Apoptosis in Non-Small Cell Lung Cancer Cells Treated With Doxorubicin Through the Inhibition of TFAP4

**DOI:** 10.3389/fgene.2019.00809

**Published:** 2019-09-10

**Authors:** Yi-Fei Wang, Xiang Ao, Ying Liu, Dan Ding, Wen-Jie Jiao, Zhuang Yu, Wen-Xin Zhai, Sheng-Hua Dong, Yu-Qi He, Hang Guo, Jian-Xun Wang

**Affiliations:** ^1^School of Basic Medical Sciences, Qingdao University, Qingdao, China; ^2^Institute for Translational Medicine, Qingdao University, Qingdao, China; ^3^Affiliated Hospital, Qingdao University, Qingdao, China; ^4^Department of Gastroenterology, The Seventh Medical Center of PLA General Hospital, Beijing, China; ^5^Department of Anesthesiology, The Seventh Medical Center of PLA General Hospital, Beijing, China

**Keywords:** microRNA-608, single nucleotide polymorphisms, apoptosis, transcription factor activating enhancer-binding protein 4, non-small cell lung cancer

## Abstract

Lung cancer is the most commonly diagnosed type of cancer and the leading cause of cancer-associated death worldwide. MicroRNAs (miRNAs) are non-coding single-stranded RNA molecules of ∼20–25 nucleotides in length. Single nucleotide polymorphisms are a class of genetic variation in the human genome, which when present in miRNA genes are associated with the risk of developing cancer. This study aimed to identify whether the miRNA (miR)-608 polymorphism rs4919510 influenced the incidence of lung cancer, and to explore the underlying mechanisms of miR-608 in the pathogenesis of the disease. A total of 37 patients with non-small cell lung cancer (NSCLC) were selected to determine the expression levels of miR-608; 96 NSCLC patients and 136 cancer-free healthy controls were recruited to determine the incidence of miR-608 rs4919510 in lung cancer patients. Additionally, the impact of miR-608 on the expression of predicted target genes, cell migration, viability, proliferation, and apoptosis was also assessed. We found that the presence of miR-608 rs4919510 did not affect the susceptibility of patients to NSCLC or the maturation of miR-608. miR-608 expression levels were found to be downregulated in NSCLC tissues. Furthermore, overexpression of miR-608 promoted doxorubicin-induced apoptosis in NSCLC cell lines A549 and HCC4006 by inhibiting the expression of transcription factor activating enhancer-binding protein 4 (TFAP4), and high expression levels of TFAP4 were observed in NSCLC tissues. Therefore, our results may provide valuable insights for the chemotherapeutical treatment of NSCLC.

## Introduction

Lung cancer is the most commonly diagnosed type of cancer worldwide, accounting for 11.6% of total cancer cases, and is also the leading cause of cancer-associated death (18.4% of total cancer cases) (Bray et al., 2018). In 2018, there were an estimated ∼234,000 new diagnoses of cancer, and 154,000 lung cancer-associated deaths (Siegel et al., 2018). Lung cancer includes small cell, as well as non-small cell lung cancer (NSCLC), of which NSCLC (including adenocarcinoma, squamous cell carcinoma, and large cell carcinoma) is the most common pathological type, accounting for ∼85% of all reported cases. Among the different types of NSCLC, adenocarcinoma is the most frequently diagnosed (Reck and Rabe, 2017). The molecular mechanism of cancer occurrence includes the inactivation of tumor suppressor genes or the abnormal activation of proto-oncogenes (Rupaimoole et al., 2016; Reck and Rabe, 2017). Although much progress has been made in determining the mechanisms of lung cancer progression, the complete picture remains uncertain. Therefore, identifying new molecules or genes associated with the occurrence, development, and diagnosis of lung cancer is paramount.

MicroRNAs (miRNAs) are non-coding single-stranded RNA molecules of ∼20–25 nucleotides, which are widely expressed in eukaryotes. Depending on their specific expression pattern, target gene expression, or cellular environment, different miRNAs are able to function as both oncogenes and tumor suppressor genes (Feng et al., 2015; Rupaimoole et al., 2016). Many miRNAs are reported to be involved in the regulation of lung cancer development, such as miRNA-31 (Edmonds et al., 2016), miRNA-135b (Lin et al., 2013), miR-886-3p (Cao et al., 2013), and miR-483-5p (Song et al., 2014).

Single-nucleotide polymorphisms (SNPs) are strongly associated with susceptibility to various diseases, including cancers. SNPs within miRNA genes may change the properties of the resulting miRNAs by altering their expression or maturation (Duan et al., 2007). The presence of SNPs in primary miRNA (pri-miRNA), precursor miRNA (pre-miRNA), and mature miRNA gene sequences can affect the regulatory network of miRNA and its subsequent influence on cell function (Duan et al., 2007; Nicoloso et al., 2010), and therefore alter the risk of cancer development. Recent studies have reported that specific miRNA polymorphisms may be associated with the risk of developing cancer (Jazdzewski et al., 2008; Sun et al., 2010; Liu et al., 2016). The relationship between miRNA SNPs and lung cancer has also been reported; miR-499 rs3746444 (Qiu et al., 2015a), miR-196a2-3p rs11614913 (Hu et al., 2008), and miR-196a2 rs11614913 (Tian et al., 2009) were significantly associated with the survival of NSCLC patients. However, elucidation of the mechanisms behind these associations requires further investigation.

miR-608 is located on human chromosome 10q24.31, and this locus also lies in an intron of the semaphorin 4G (SEMA4G) gene. Current studies indicate that miR-608 is downregulated in some malignant tumors and can inhibit cell proliferation, invasion, and migration, or promote apoptosis (Wang et al., 2016b; Yang et al., 2016; Liang et al., 2017). The rs4919510 variant G allele of miR-608 is one of the most important miRNA-related SNPs reported to date. miR-608 rs4919510 was reported to be associated with altered risk of developing colorectal cancer (Ying et al., 2016), breast cancer (Huang et al., 2012), papillary thyroid cancer (Wei et al., 2015), nasopharyngeal carcinoma (Qiu et al., 2015b), and lung cancer (Yin et al., 2016). However, there are conflicting results for the association between the presence of miR-608 rs4919510 and susceptibility to tumors. Other studies have suggested that rs4919510 is not associated with the incidence of breast (Dai et al., 2016) and colorectal cancer (Ranjbar et al., 2018). Therefore, further research is required to clarify the relationship between miR-608 rs4919510 and susceptibility to lung cancer.

Transcription factor activating enhancer-binding protein 4 (TFAP4) is an important regulator in the genesis and progression of human cancers. It is widely expressed in human tissues and was first discovered by Mermod et al. (1988) in the enhancer binding factor of *in vitro* transcription of the viral SV40 late protein (Atchley and Fitch, 1997). TFAP4 has been shown to promote invasion and metastasis in certain types of cancer cell (Butter et al., 2012; Ma et al., 2018; Wu et al., 2018). It has also been reported to promote tumorigenic capability by activating the Wnt/β-catenin pathway in hepatocellular carcinoma (Song et al., 2018). However, there are few reports on TFAP4 and tumor cell apoptosis; thus, the present study aimed to investigate the potential relationship between TFAP4 and apoptosis.

In this report, we also aimed to identify the effects of the miR- 608 rs4919510 polymorphism on the incidence of lung cancer, and to elucidate the underlying mechanisms of miR-608 in the pathogenesis of the disease. We demonstrated that miR-608 rs4919519 did not affect the incidence of NSCLC or the processing of miR-608. We also showed that miR-608 expression levels were downregulated in NSCLC tissues. Furthermore, overexpression of miR-608 promoted doxorubicin (DOX)-induced apoptosis in A549 and HCC4006 cells by targeting TFAP4. Taken together, our data suggested that miRNA-608 promoted apoptosis in NSCLC cells treated with DOX through the inhibition of TFAP4.

## Methods

### Study Subjects

Blood samples were collected from 96 NSCLC patients and 136 cancer-free controls, and genotyping was conducted to determine the incidence of the miR-608 rs4919510 polymorphism. We subsequently collected 37 sets of paired NSCLC and non-malignant tissues to detect the expression of miR-608 and TFAP4. None of these patients had previously received chemotherapy or radiotherapy. All clinical samples were collected at the Affiliated Hospital of Qingdao University (Qingdao, China). All subjects were of the Han Chinese population, from Qingdao and the surrounding areas, and were not related to each other.

### Cell Lines and Treatments

Human lung adenocarcinoma A549 cells, HCC4006 cells, and HEK-293 cells were obtained from our personal laboratory stock. HEK-293 cells were cultured in Dulbecco’s modified Eagle’s medium (Gibco; Thermo Fisher Scientific, Inc.). A549 and HCC4006 cells were cultured in RPMI 1640 (Gibco; Thermo Fisher Scientific, Inc.). All media was supplemented with 10% fetal bovine serum, 100 U/ml penicillin, and 100 mg/ml streptomycin (Gibco; Thermo Fisher Scientific, Inc.), and cells were cultured in a humidified atmosphere containing 5% CO_2_ at 37˚C. Cells were treated with 2 or 0.2 µM DOX unless indicated otherwise.

### Genotyping

Genotyping was performed using the polymerase chain reaction (PCR) and DNA sequencing. Genomic DNA was extracted using the DNA Blood Mini Kit (Qiagen, Inc.), according to the manufacturer’s protocol. The genomic DNA was used as a template, and the target bands were amplified using PCR; successful genotyping was confirmed by DNA sequencing. The PCR primers used to amplify the rs4919510 C > G site in the miR-608 precursor are shown in **Supplementary Table 2**.

### Construction of miR-608 Expression Vectors

Vectors coding for the pri-miR-608 C and G allele were constructed separately. Following genotyping, DNA fragments (∼487 bp) containing the miR-608 precursor sequence were amplified from human genomic DNA and cloned into the pcDNA3.1 vector (Invitrogen; Thermo Fisher Scientific, Inc.) using the Trelief™ soSoo Cloning Kit Ver.2 (Tsingke Company) following *Eco*RI restriction endonuclease digestion. The primer sequences are shown in **Supplementary Table 2**. Direct sequencing was used to confirm the vector sequences, which differed only at the SNP rs4919510 site.

### RNA Extraction and Reverse Transcription-Quantitative PCR (RT-qPCR)

In the present study, stem-loop RT-qPCR and poly (A) tailing-based RT-qPCR were conducted. Total RNA was extracted using TRIzol^®^ reagent (Invitrogen, Carlsbad, CA). RNA used to detect the expression level of pre-miR-608, and TFAP4 was reversely transcribed with a reverse transcriptase kit from Takara Bio, Inc.; RNA used to detect the expression level of mature miR-608 was reversely transcribed with the Mir-X miRNA qRT-PCR SYBR^®^ Kit (Takara Bio, Inc.). cDNA amplification was conducted using the SYBR Premix Ex TaqII kit (Takara Biomedical Technology) according to the manufacturer’s instructions. U6 and GAPDH were used as internal reference genes to detect the relative expression levels of mature and pre-miR-608, and TFAP4, respectively. The primers used for the RT-PCR reactions are shown in **Supplementary Table 2**.

### Transfection With miRNA and Small Interfering RNAs (siRNAs)

The miRNA mimic, var mimic, and inhibitor were synthesized by Shanghai GenePharma Co., Ltd. The corresponding RNA sequences were as follows: miR-608 mimic, 5’-AGGGGUGGUGUUGGGACAGCUCCGU-3’; miR-608 var mimic 5’-AGGGGUGGUGUUGGGACAGCUGCGU-3’; and miR-608 inhibitor, 5’- ACGGAGCUGUCCCAACACCACCCCU-3’. siRNA targeting human TFAP4 (siTFAP4) was also synthesized by Shanghai GenePharma Co., Ltd., for which the sequence was 5’-GACGCAUGCAGAGCAUCAATT-3’. Cells were transfected with miRNA mimic (100 nmol/l), inhibitor (100 nmol/l), or siRNA (150 nM) using Lipofectamine^®^ 2000 (Invitrogen; Thermo Fisher Scientific, Inc.) according to the manufacturer’s protocol. Briefly, A549 and HCC4006 cells were seeded into tissue culture plates at 40–60% confluency, 24 h prior to use; cells were then transfected with siRNA or miRNA according to the manufacturer’s instructions. The transfected cells were harvested 24 and 48 h post-transfection for RNA or protein detection, respectively, unless otherwise indicated. All transfections were carried out in triplicate.

### MTT Assay

A549 and HCC4006 cells were seeded into 96-well plates at a density of 1 × 10^4^ cells/well. When the cells had adhered, they were transfected with the aforementioned miRNA or siRNA. Cells were incubated for 24, 48, 72, and 96 h, and 20 μl MTT solution was added to each well. After a further 4-h incubation, the culture medium was aspirated and 100 μl DMSO was added to each well to dissolve the formazan crystals. The plates were then shaken for 5 min at room temperature, and the absorbance was determined using micro-plate reader at a wavelength of 490 nm (SpectraMax i3, Molecular Devices, LLC).

### Wound Healing Assay

A549 and HCC4006 cells were seeded into six-well plates (5 × 10^5^ cell/well) and transfected with the aforementioned miRNA or siRNA reagents. The cell monolayer was scratched with a 200-μl pipette tip 6 h post-transfection and incubated for a further 48 h. Images were captured at 0, 24, and 48 h post-scratch, and ImageJ software (National Institutes of Health) was used to determine the scratch healing area. The analysis was performed for all experimental variants, and three independent experiments were performed in triplicate.

### Transwell Assay

A549 and HCC4006 cells were seeded into six-well plates at a density of 1–10 × 10^5^ cells/well and transfected with the aforementioned miRNA or siRNA reagents as above. After a 24-h incubation period, the cells were harvested and resuspended in serum-free medium; 500 µl medium (10% FBS) was added to each well of a 24-well plate, in addition to a Transwell chamber. Then, 100 µl serum-free medium was added to the Transwell chamber in addition to 3 × 10^4^ cells. Following a 24 h incubation period, cells on the surface of the upper membrane were removed and the lower membrane surface was fixed with 4% paraformaldehyde. Cells were washed twice with PBS and stained with 0.5% crystal violet for 30 min. The cells were subsequently counted in ≥5 random fields using an optical microscope (magnification, ×100 and ×200).

### Apoptosis Assay

DOX is a DNA-intercalating agent that is widely used as a first-line clinical therapeutic to treat a variety of malignancies, including lung cancer (Calvo et al., 2017; Zhou et al., 2018). A549 and HCC4006 cells were seeded into six-well plates at a density of 1 × 10^5^ cells/well, transfected with miRNA, and treated with DOX (2 or 0.2 μM) for 24 h. The cells were washed twice using PBS and subsequently harvested. Cells were centrifuged to remove the supernatant, and 400 μl binding Buffer was added prior to staining with of Annexin V and propidium iodide (PI) according to the manufacturer’s instructions of cell apoptosis detection kit (Majorbio Biotech, Shanghai, China). Apoptosis was detected using the FACS Calibur system (BD Biosciences).

### Cell Death

Cell death was determined using Trypan blue exclusion method. Trypan blue-positive cells were counted using a hemocytometer (Shanghai Anxin Optical Instrument Manufacture Co., Ltd.). Cells (150) were randomly selected in the field of view to calculate cell death rate.

### Bioinformatics

Target biogene prediction of miR-608 was performed using TargetScan Human 7.2 bioinformatics prediction software (http://www.targetscan.org/vert_72/). TargetScan predicts the biological targets of miRNAs by searching for the presence of conserved 6, 7, and 8mer sites that match the seed region of each miRNA (Lewis et al., 2005). We used the TargetScan context scores to assess per gene and miRNA. The miRNAs targeting TFAP4 were selected based on a TargetScan context score cut-off of ≤0.01.

Kaplan–Meier–Plotter analysis (http://kmplot.com/analysis/) was used to evaluate the relationship between miR-608 or TFAP4 and prognosis in patients with NSCLC.

### Luciferase Reporter Assay

We synthesized one wild-type and three mutant fragments (Mut 1, Mut 2, and Mut 1 + 2) of the 3′UTR of the human TFAP4 gene, which contains putative binding sites for miR-608. The fragments were then cloned into the pGL3-control reporter vector (Promega Corporation). TFAP4 3’UTR-Mut 1 vector contained mutated site 1 and wild-type site 2. TFAP4 3’UTR-Mut 2 vector contained wild-type site 1 and mutated site 2. TFAP4 3’UTR-Mut 1 + 2 vector contained mutated site 1 and mutated site 2. Mut 1 and Mut 2 vectors have 4 bases changed in the 3’UTR miR-608 binding sites, whereas TFAP4 3′UTR-Mut 1 + 2 contained 8 bases changed in the 3’UTR miR-608 binding sites. Using Lipofectamine^®^ 2000 (Invitrogen), a total of 200 ng/well luciferase reporter constructs, 5 ng/well pRL-TK vector, and 400 ng/well miR-608 mimic, miR-608, var mimic or mimic control were co-transfected into HEK-293 cells seeded into 24-well (1 × 10^4^ cells/well). Forty-eight hours post-transfection, the cells were collected and the luciferase *Renilla* luciferase activities were detected using a dual luciferase kit (Promega Corporation) according to the manufacturer’s instructions.

### Western Blotting

Western blotting was performed to determine the expression level of TFAP4 and cleaved caspase-3. The total protein of the cell samples was extracted using radioimmunoprecipitation assay buffer (Beijing Solarbio Science & Technology Co., Ltd.) containing a protease inhibitor cocktail. Total protein was quantified using a BCA Protein assay kit (Beyotime Institute of Biotechnology). Proteins samples were separated using SDS-PAGE on a 12% gel and transferred to nitrocellulose membranes. After blocking for 1 h with 5% non-fat milk, the membranes were incubated with primary antibodies, including anti-TFAP4 (1:1,000), anti-cleaved caspase-3 (1:1,000), and anti-β-actin (1:2,000) at 4˚C overnight. The membranes were then incubated for an additional hour at room temperature with the following secondary antibodies; horseradish peroxidase-conjugated (HRP) goat anti-rabbit IgG (1:2,000) and HRP-conjugated goat anti-mouse IgG (1:2,000). Signals were detected using an enhanced chemiluminescence system (Pierce; Thermo Fisher Scientific, Inc.) according to the manufacturer’s protocol and exposed using X-ray film (Kodak). Anti-TFAP4 was purchased from ABclonal Biotech Co., Ltd. Anti-cleaved caspase-3, anti-β-actin, HRP-conjugated goat anti-rabbit IgG and HRP-conjugated goat anti-mouse IgG were purchased from Cell Signaling Technology, Inc.

### Statistical Analysis

The experimental data was analyzed using SPSS17.0 software (SPSS, Inc.), and the results were expressed as the means ± standard deviation of at least three independent experiments. The two-tailed Student’s t-test was used for comparisons between two groups, and one-way ANOVA was used for comparisons among ≥3 groups. Mann–Whitney test (non-parametric test) was used to analyze the data from unpaired samples that are not normal distribution. P < 0.05 was considered to indicate a statistically significant difference.

## Results

### The Presence of the miR-608 rs4919510 Polymorphism Is Not Associated With the Incidence of NSCLC

In the present study, we explored whether miR-608 was associated with the incidence of lung cancer. A total of 96 NSCLC patients and 136 cancer-free healthy controls were genotyped to determine the frequency of the miR-608 rs4919510 polymorphism. Peripheral blood was taken from patients immediately after admission. As shown in **Table 1**, the group adheres to the Hardy–Weinberg equilibrium. The results revealed that the rs4919510 (C > G) polymorphism was not associated with an increased risk of developing NSCLC. In the total population, the GG genotype was represented in 33.8% of the NSCLC controls, and 33.3% of cases, corresponding to an adjusted odds ratio (OR) of 1.208 [95% confidence interval (CI), 0.587–2.489]; the CG genotype was present in 41.9% of controls and 47% of cases, with an OR of 1.371 (95% CI, 0.69–2.724). Compared with the C allele, the G allele was present in 54.8% of the controls and 56.8% of NSCLC cases, with an OR of 1.084 (95% CI, 0.747–1.573). We performed meta-analyses to confirm the association of the miR-608 rs4919510 (C > G) with lung cancer risk. The results showed that no association was observed between the presence of miR-608 rs4919510 (C > G) and the risk of developing lung cancer, suggesting that the miR-608 rs4919510 (C > G) polymorphism has no association with lung cancer risk in the Chinese Han population.

**Table 1:** MiR-608 rs4919510 polymorphism has no relation with the incidence of NSCLC.

Genotypes/allele types Rs4919510	Controls (N = 136)	Lung cancer patients (N = 96)	OR (95%CI)	P-value
	N (%)	N (%)		
**CC**	33 (24.3)	19 (19.7)	1.00	
**CG**	57 (41.9)	45 (47)	1.371 (0.69, 2.724)	0.367
**GG**	46 (33.8)	32 (33.3)	1.208 (0.587, 2.489)	0.608
**C**	123 (45.2)	83 (43.2)	1.00	
**G**	149 (54.8)	109 (56.8)	1.084 (0.747, 1.573)	0.608

### miR-608 Is Downregulated in Human NSCLC Tissues

**Figure 1:**
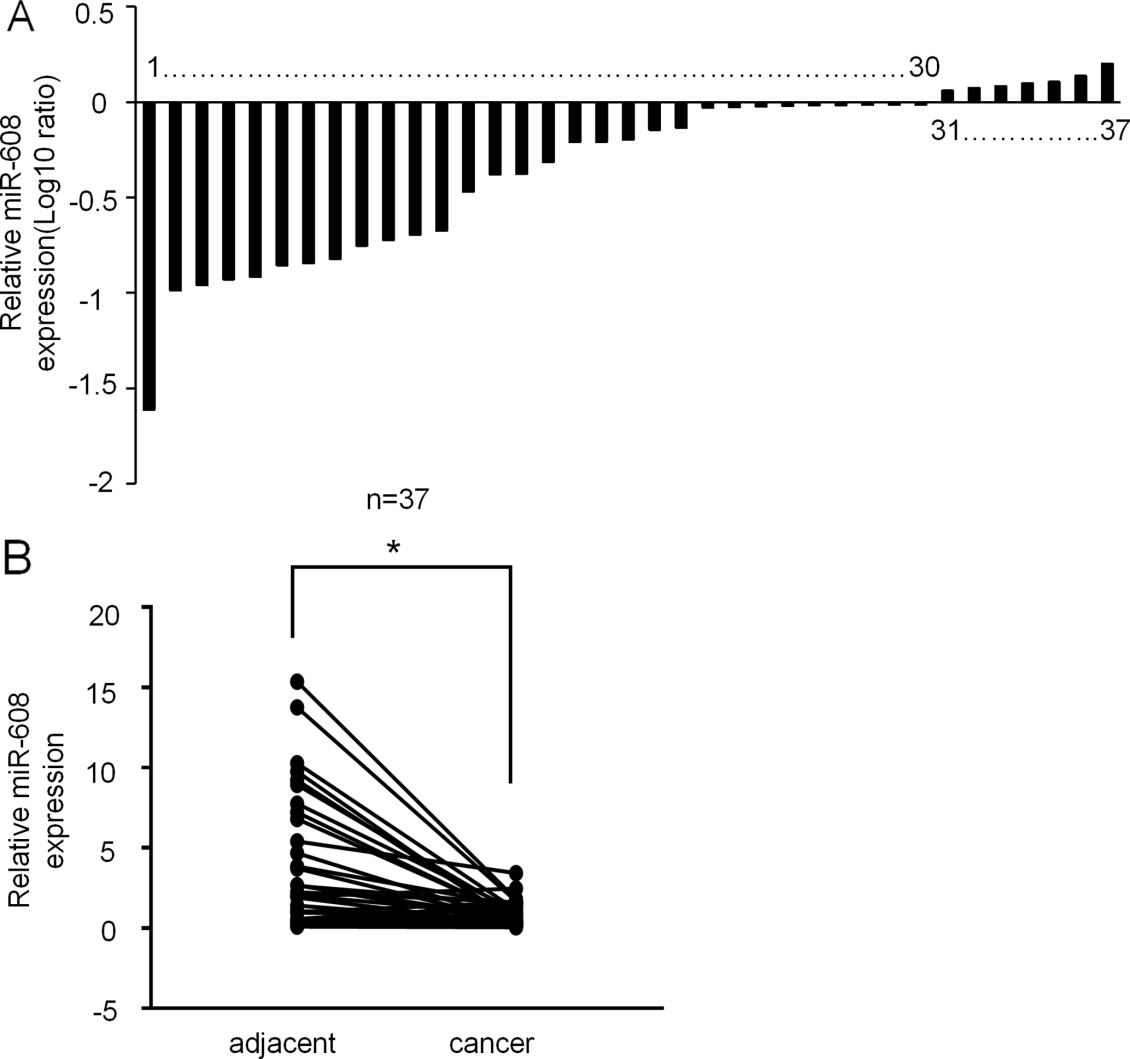
Expression of miR-608 in human NSCLC tissues. **(A)** Result showing the relative expression level of miR-608 (log10 ratio) in 37 pairs of samples (NSCLC tissues and matched normal tissues) by RT-qPCR. **(B)** Comparison of miR-608 expression in NSCLC tissues and matched normal tissues analyzed by non-parametric test (n = 37). For comparison, the expression level of miR-608 in matched normal tissue of NSCLC patient 1 was set to 1; *P < 0.05. miR, microRNA; NSCLC, non-small cell lung cancer.

Given the limited understanding of the role of miR-608 in lung cancer, we determined the miR-608 expression levels in a panel of human NSCLC tissues using RT-qPCR. miR-608 expression was confirmed in NSCLC tissues and pair-match adjacent normal lung tissues from 37 NSCLC patients; the tumor and patient information is presented in **Supplementary Table 1**. As shown in **Figures 1A, B**, the expression level of miR-608 was significantly decreased in the tumor tissues of 81% (30/37) of the NSCLC patients, compared with their matched adjacent normal tissues (P < 0.05). In addition, we found that low expression of miR-608 was significantly correlated with the gender of NSCLC patients (**Table 2**), whereas not correlated with the prognosis of NSCLC patients (**Supplementary Figure 1**). Based on these findings, we speculated that miR-608 might act as tumor suppressor gene in the development and progression of NSCLC.

**Table 2:** Association between the miR-608 and TFAP4 expression and clinicopathological characteristics of NSCLC patients.

Characteristics	has-miR-608		P-value	TFAP4		P-value
	Low-expression	High-expression		Low-expression	High-expression	
Total	18	19		18	19	
Age			0.124			0.176
>60	11	16		15	12	
≤60	7	3		3	7	
Gender			0.037*			0.603
Male	7	14		11	10	
Female	11	5		7	9	
Tumor size			0.293			0.969
≥3 cm	0	2		1	1	
<3 cm	18	17		17	18	
Clinical stage			0.404			0.969
I	12	15		16	11	
II–IV	6	4		2	8	
Lymphatic metastasis			0.586			0.046*
Yes	1	2		0	3	
No	17	17		18	16	
Metastasis			0.969			0.184
M0	17	18		18	17	
M1	1	1		0	2	

### rs4919510 Does Not Affect the Maturation of miR-608

**Figure 2:**
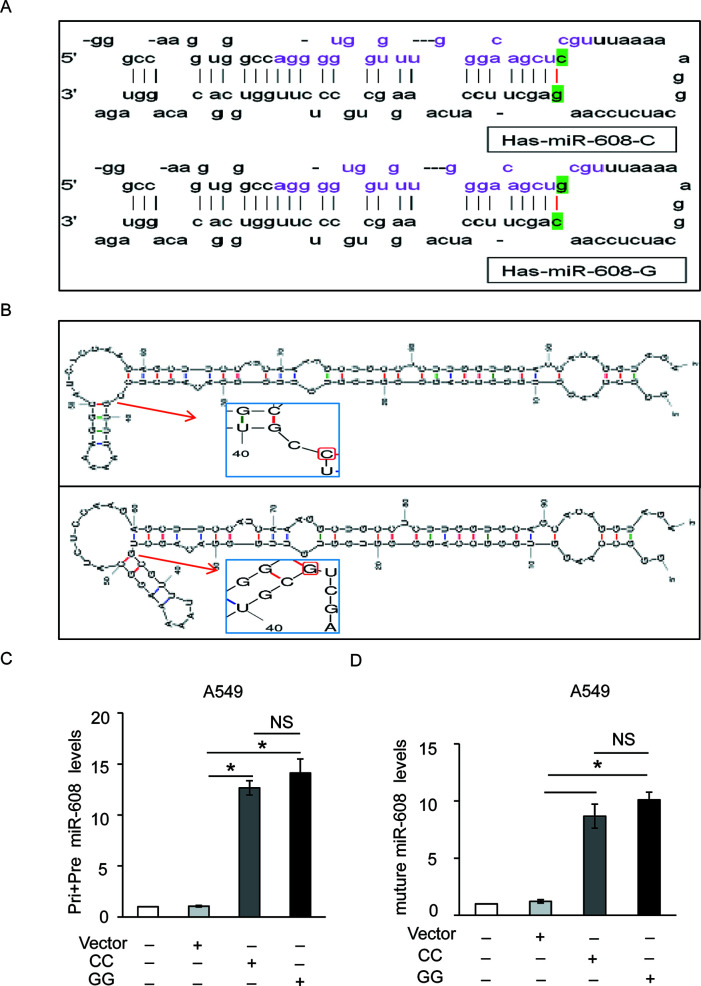
Effect of miR-608 rs4919510 polymorphism on miR-608 maturation. **(A)** Hairpin loop structure of the miR-608 precursor including C- and G-allelic variants. The red color in the mature miR-608 sequence corresponds to the rs4919510 C to G polymorphism. **(B)** Secondary structure of pre-miR-608-C and pre-miR-608-G predicted using the mfold web server. RT-qPCR results showed the effect of rs4919510 C to G polymorphism on maturation of pri, pre-miR-608 **(C)**, and mature miR-608 **(D)** in A549 cells. *P < 0.05. NS, No significance; miR, microRNA; pri-miRNA, primary miRNA; pre-miRNA, precursor miRNA.

Our results suggested that the miR-608 rs4919510 polymorphism did not influence susceptibility to NSCLC. SNPs may change the properties of miRNAs by altering miRNA expression and/or maturation. To explore the functionality of the rs4919510 G/C polymorphism, we further validated the potential effects of the G/C variant on the expression of mature miR-608. As shown in **Figure 2A**, the rs4919510 polymorphism is located at the 37^th^ position of pre-miR-608 sequence (the 4th position of miR-608-3p). Results from miRBase revealed that this genetic variant resulted in a G:C to C:G switch in the stem loop structure of the miR-608 precursor, which strongly interfered with loop formation within the 3’ region of the mature miR-608. Using mfold software, we predicted the secondary structure of miR-608-C and miR-608-G mfold to assess whether the secondary structure of miR-608 was altered by this nucleotide substitution. We determined that the secondary structure of miR-608 did change as a result of this C to G substitution (**Figure 2B**). The optimal free energy was also reduced from −31.1 kcal/mol for the C allele to −30.3 kcal/mol for the G allele, though this was not particularly obvious. Therefore, we aimed to verify whether this change affected the maturation of miR-608 in A549 and HCC4006 cells. The precursor miR-608 containing the G or C allele was cloned into the pcDNA3.1 vector and transfected into A549 and HCC4006 cells. miRNAs are initially transcribed as pri-miRNAs, which are then processed into ∼65 nt hairpin-shaped pre-miRNAs. To determine which step during miRNA biogenesis may have been altered by the rs4919510 polymorphism, we performed RT-qPCR to determine the relative expression levels of the mature miR-608 alone or in addition to those of pri-miR-608 and pre-miR-608 in transfected cells. The results showed that there was no significant difference in the expression level of miR-608-C and miR-608-G at the level of pri-miRNA + pre-miRNA combined (**Figure 2C** and **Supplementary Figure 2A**) or the mature miRNA (**Figure 2D** and **Supplementary Figure 2B**) level in two NSCLC cell lines. These data indicated that the rs4919510 polymorphism did not alter the processing of pri- miR-608 to mature miR-608.

### miR-608 Promotes DOX-Induced Apoptosis in NSCLC Cells

**Figure 3:**
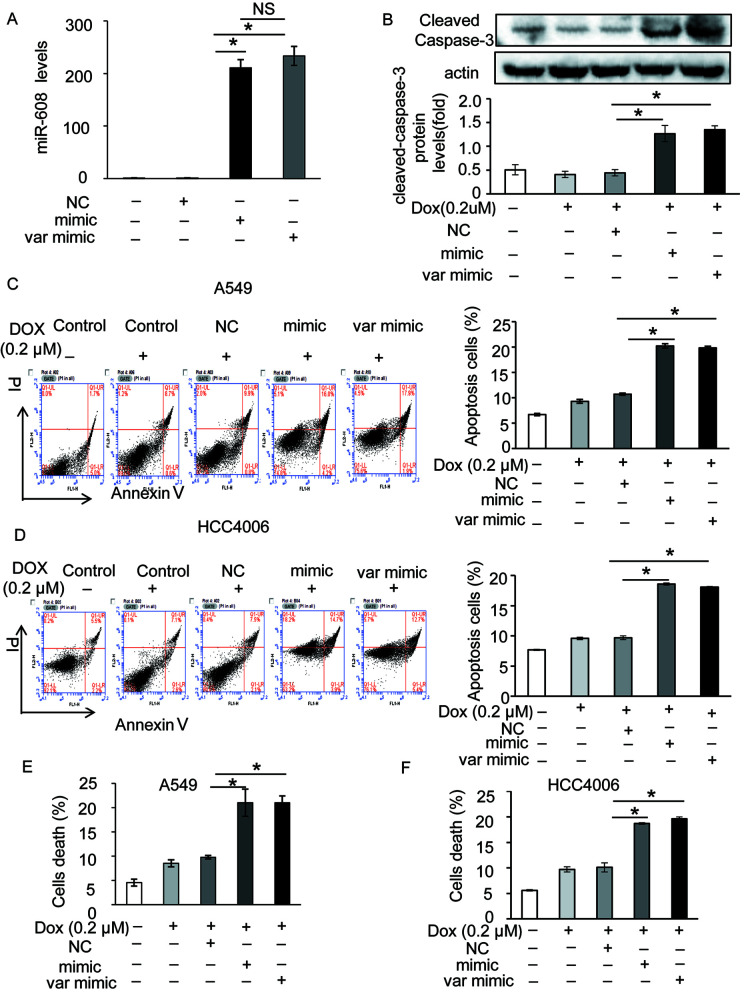
Effect of miR-608 overexpression on DOX-induced apoptosis in NSCLC cells. **(A)** The expression level of miR-608 in A549 cells transfected with miR-608 mimic or var mimic determined by RT-qPCR. NS, No significance. **(B)** The expression level of cleaved capase-3 in miR-608-overexpressed A549 cells treated with or without low-dose DOX (0.2 μM) determined by Western blot. Apoptosis was determined by propidium iodide/Annexin-V double-staining in A549 **(C)** and HCC4006 **(D)** cells. Cell death was determined by trypan blue staining in A549 **(E)** and HCC4006 **(F)** cells. *P < 0.05.

**Figure 4:**
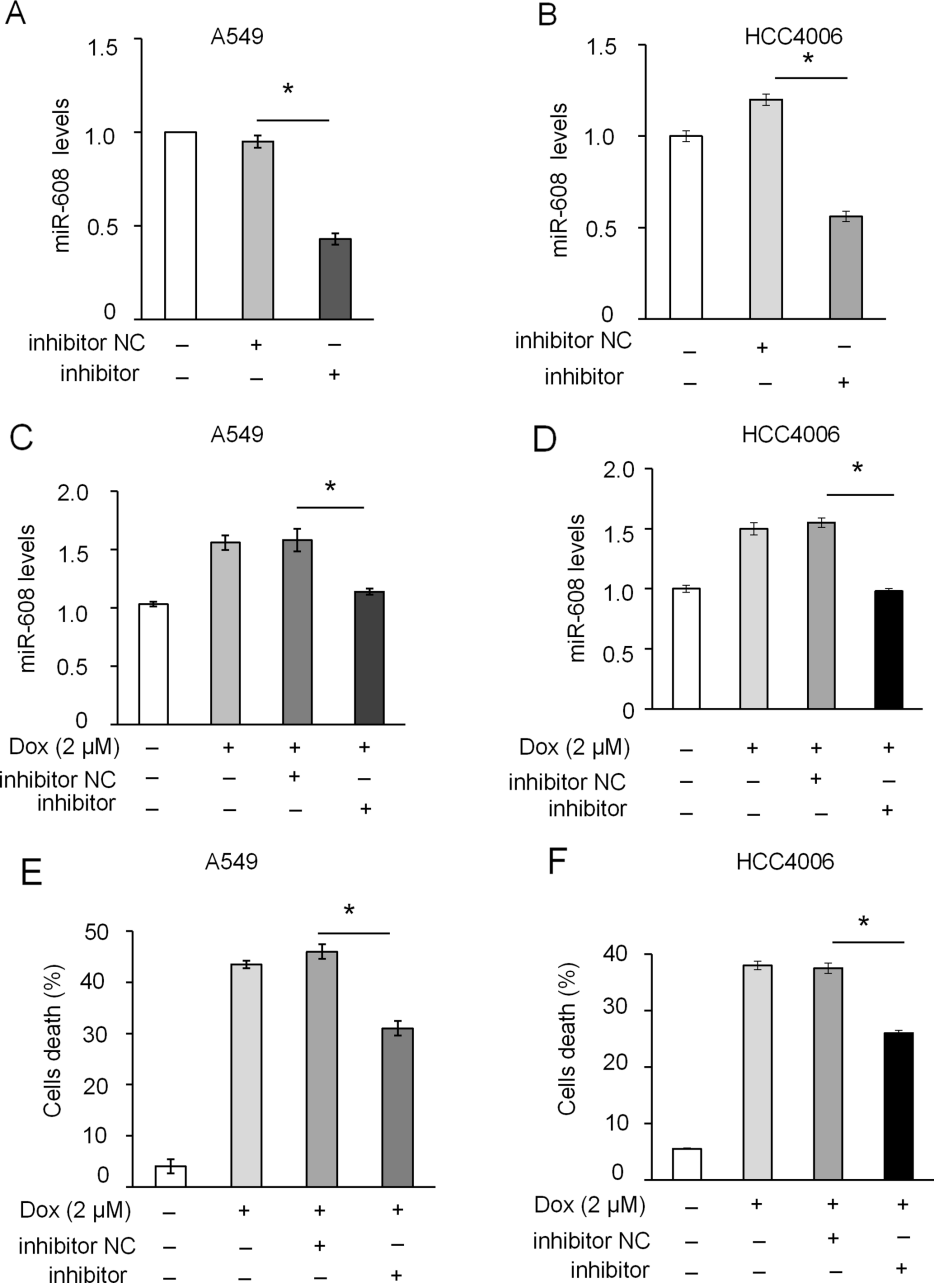
Effect of miR-608 inhibition on DOX-induced apoptosis in NSCLC cells. RT-qPCR results showing the inhibition of endogenous miR-608 expression by miR-608 inhibitor in A549 **(A)** and HCC4006 **(B)** cells. RT-qPCR results showing the expression of miR-608 in DOX-induced (2 μM) A549 **(C)** and HCC4006 **(D)** cells transfected with miR-608 inhibitor. Trypan blue staining results showing the inhibition of cell death in DOX-induced A549 **(E)** and HCC4006 **(F)** cells. Each experiment was independently repeated ≥3 times. The data are presented as the mean ± standard deviation. Student’s t-test, *P < 0.05. miR, microRNA; DOX, doxorubicin.

The rs4919510 polymorphism is in the 4th position of the miR-608-3p sequence. To determine whether the polymorphism alters the function of miR-608 in NSCLC cells, we synthesized an exogenous miR-608-C mimic and miR-608-G var mimic to enhance the expression of miR-608 in A549 and HCC4006 cells (**Figure 3A** and **Supplementary Figure 3A**). We found that the overexpression of miR-608 had no significant effect on the apoptosis of A549 (**Supplementary Figure 3B**) and cell death of A549 (**Supplementary Figure 3C**) and HCC4006 cells (**Supplementary Figure 3D**). We also intended to elucidate the effect of miR-608 on apoptosis following DOX treatment. The apoptosis cell ratio induced by low-dose DOX administration (0.2 μM) was markedly decreased to <10%. However, overexpression of miR-608 in A549 cells treated with low-dose DOX resulted in a significant increase in the protein expression level of cleaved-caspase-3 (**Figure 3B**), in addition to an increase in apoptosis in A549 and HCC4006 cells (**Figures 3C, D**). Moreover, overexpression of miR-608 also significantly increased DOX-induced cell death in A549 and HCC4006 cells (**Figures 3E, F**). This demonstrated that the overexpression of miR-608 sensitized A549 and HCC4006 cells to DOX treatment, and miR-608 may serve a pro-apoptotic role in NSCLC. Furthermore, we observed that both the miR- 608 mimic and var mimic markedly increased DOX-induced apoptosis. To clarify the role of endogenous miR-608 in DOX-induced apoptosis, A549 and HCC4006 cells were transfected with miR-608 inhibitor to prevent its expression (**Figures 4A, B**). We observed that the upregulated expression of miR-608 (**Figures 4C, D**), and the cell death induced by DOX (2 μM) was attenuated by inhibiting miR-608 expression (**Figures 4E, F**), suggesting that miR-608 may mediate DOX-induced apoptosis. Taken together, these results suggested that miR-608 is able to promote apoptosis following treatment with DOX, and that the miR-608 rs4919510 polymorphism may have the same effect on its function in apoptosis.

**Figure 5:**
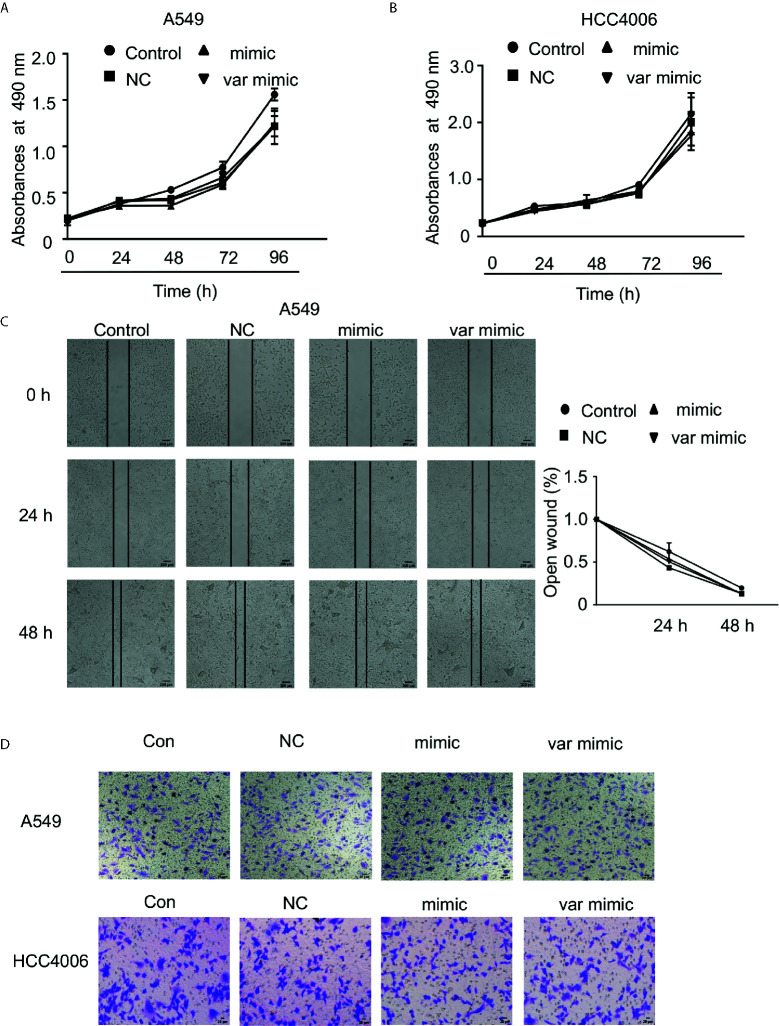
Effect of miR-608 on the proliferation and metastasis of NSCLC cells. Cell proliferation was examined by MTT assay in A549 **(A)** and HCC4006 **(B)** cells transfected with miR-608 mimic or var mimic for the indicated times. **(C)** The effect of miR-608 on metastasis determined by wound-healing assay in A549 cells. Transwell assay results showing the effect of miR-608 on metastasis in A549 cells **(D)** and HCC4006 **(E)** cells. miR, microRNA.

Additionally, we wanted to verify if miR-608 rs4919510 polymorphism influenced the proliferation and migration of NSCLC cell lines. We found that neither the miR-608 mimic nor the var mimic influenced cell proliferation (**Figures 5A, B**) or migration (**Figures 5C–E**) in A549 and HCC4006 cells.

### mir-608 Directly Targets TFAP4

**Figure 6:**
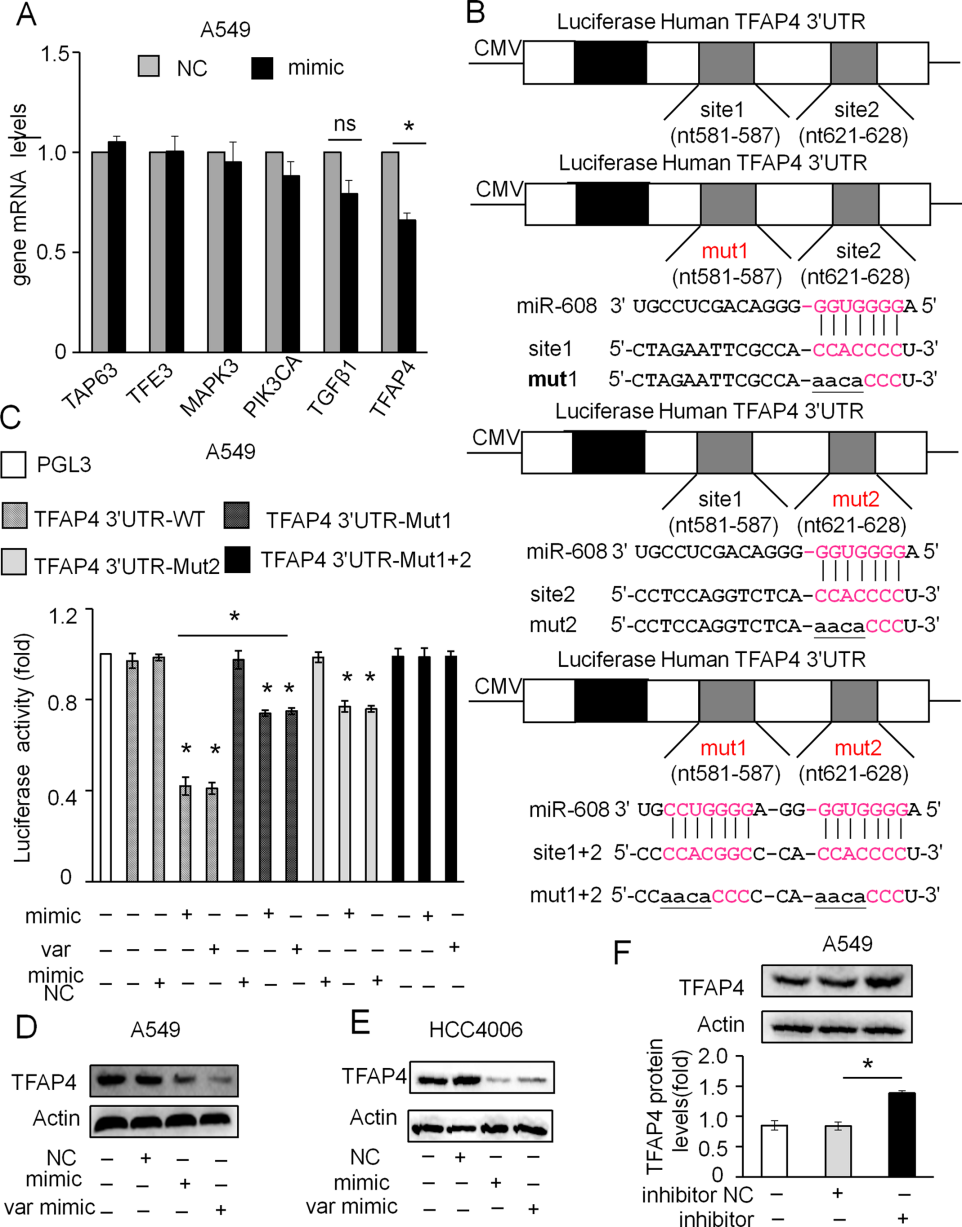
miR-608 directly targets TFAP4. **(A)** Effect of miR-608 overexpression on mRNA levels of *TAP63*, *TFE3*, *MAPK3*, *PIK3CA*, *TGF*β*1*, and *TFAP4* determined by RT-qPCR in A549 cells. **(B)** Binding sites of the wild-type and three mutants of the TFAP4 3′UTR, with the mutant sites labeled in lowercase letters and underlined. **(C)** Effect of miR-608 overexpression on luciferase activity in HEK-293 cells transfected with distinct TFAP4 3′UTR reporters (n = 3). Effect of miR- 608 overexpression on the expression of TFAP4 determined by Western blot in A549 cells **(D)** and HCC4006 **(E)** cells. Effect of miR-608 inhibition on the expression of TFAP4 in A549 cells **(F)**. The data is presented as the mean ± standard deviation. Student’s t-test, *P < 0.05. miR, miRNA; TFAP4, transcription factor activating enhancer-binding protein 4; TAP63, tumor protein p63; TFE3, transcription factor binding to IGHM enhancer 3; MAPK3, mitogen-activated protein kinase 3; PIK3CA, phosphatidylinositol-4,5-bisphosphate 3-kinase catalytic subunit alpha; TGFβ1, transforming growth factor β1.

TargetScan software was used to predict the downstream target genes of miR-608. Eight genes that may interact with miR-608 were found. Among these genes, *BCL2L1* (Othman et al., 2013; Choi et al., 2015) and *AK*T (Liang et al., 2017; Othman and Nagoor, 2017) have been reported to be the target genes of miR-608. Next, we detected the effect of miR-608 overexpression on the expression of *TP63*, *TFAP4*, *TFE3*, *TGFβ1*, *MAPK3*, and *PIK3CA* in A549 cells. The results showed that miR-608 overexpression only significantly decreased the expression of mRNA, whereas it did not significantly affect the expression of other potential target genes, indicating that *TFAP4* is a direct target gene of miR-608 (**Figure 6A**). We used the TargetScan context scores to assess the binding of the miRNA to the target gene. The miRNAs targeting TFAP4 were selected based on a TargetScan context score cutoff of ≤0.01, as we hypothesized that TFAP4 might be a potential target gene of miR-608. Wild type TFAP4 possesses two potential binding sites for miR-608 in its 3′UTR: site 1 (nt 581–587) and site 2 (nt 621–628). We constructed a wild-type luciferase reporter gene vector (TFAP4 3’UTR-WT) and three mutated luciferase constructs to detect whether miR-608 directly participates in the regulation of TFAP4. **Figure 6B** shows the introduction of the mutations. Luciferase reporter assay revealed that miR-608 overexpression significantly decreased the luciferase activity in cells transfected with TFAP4 3’UTR-WT compared with that in cells transfected with TFAP4 3′UTR-Mut 1 + 2. Moreover, overexpression of miR-608 also reduced the luciferase output of TFAP4 3′UTR-Mut 1 and TFAP4 3′UTR-Mut 2 in a small, but statistically significant manner compared with the reduction caused by TFAP4 3’UTR-WT (**Figure 6C**), indicating that the two separate binding sites in TFAP4 3’UTR were able to bind to miR-608. These results demonstrated that miR-608 directly binds to TFAP4 3′UTR through the two separate binding sites, and is thus able to regulate the expression of TFAP4. We further found that miR-608 is able to suppress TFAP4 expression in A549 and HCC4006 cells (**Figures 6D, E**). Meanwhile, miR-608 inhibition led to upregulation of TFAP4 in A549 cells (**Figure 6F**). Collectively, these data showed that *TFAP4* is a downstream target gene of miR-608, which may be involved in miR-608-mediated regulatory networks in lung cancer.

### miR-608 Regulates DOX-Induced Apoptosis by Targeting TFAP4

TFAP4 has been reported to be upregulated in multiple tumor types, including colorectal (Yang et al., 2018) and gastric carcinoma (Wu et al., 2018), which suggests that TFAP4 has an oncogenic role in cancer pathogenesis and progression. We have previously determined that miR-608 can suppress the expression of TFAP4, and therefore speculated that TFAP4 may influence the miR-608-regulated DOX sensitivity of A549 and HCC4006 cells.

**Figure 7:**
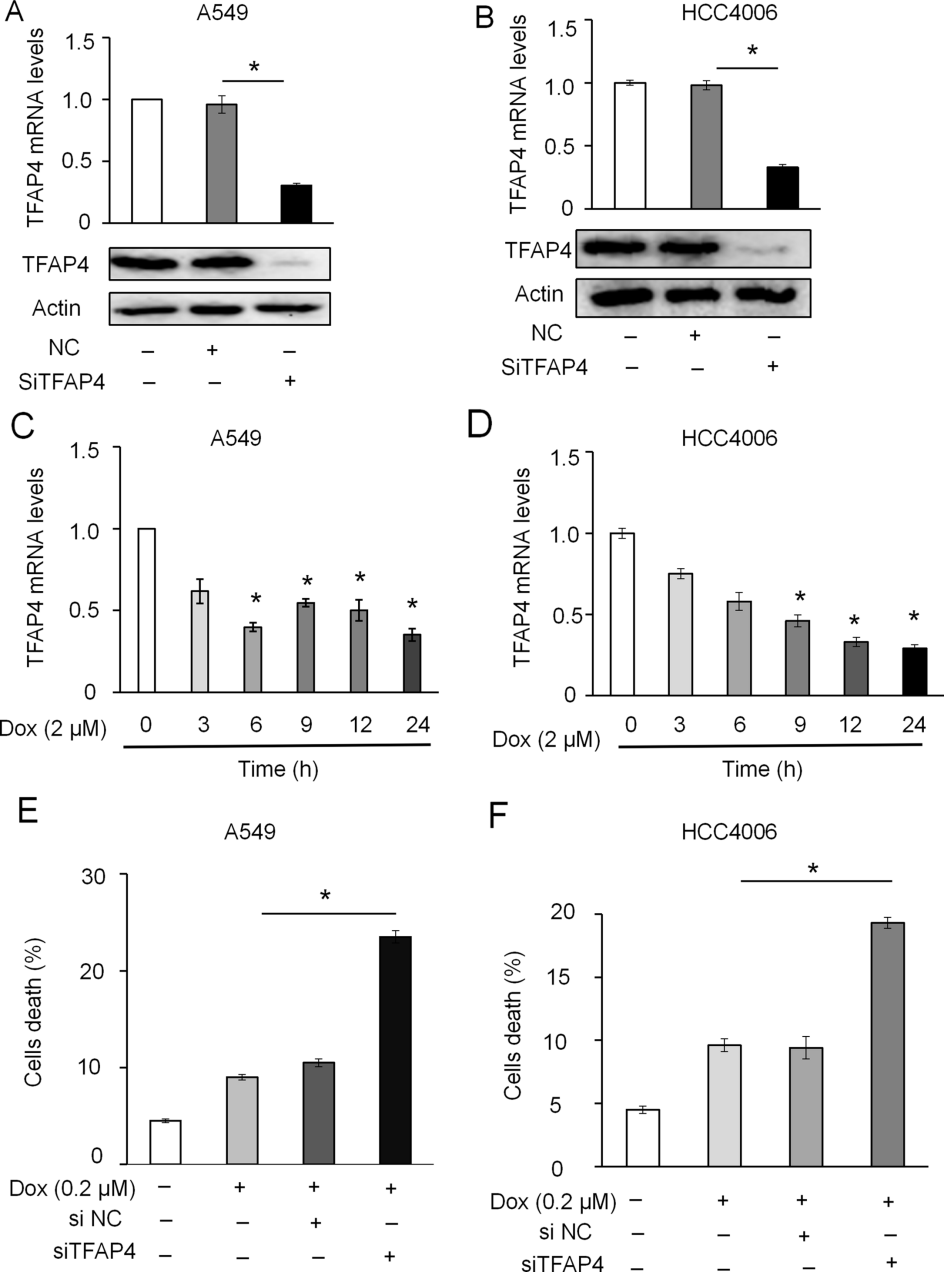
Effect of TFAP4 knockdown on DOX-induced apoptosis in NSCLC cells. Expression levels of TFAP4 were detected using RT-qPCR and Western blot in A549-TFAP4 knockdown cells **(A)** and HCC4006-TFAP4 knockdown cells **(B)**. Expression level of TFAP4 mRNA in A549 **(C)** and HCC4006 cells **(D)** treated with DOX (2 μM) or the indicated times. Effect of low-dose DOX (0.2 μM) on cell death of A549 **(E)** and HCC4006 cells **(F)** transfected with or without siTFAP4. Student’s t-test, *P < 0.05.

**Figure 8:**
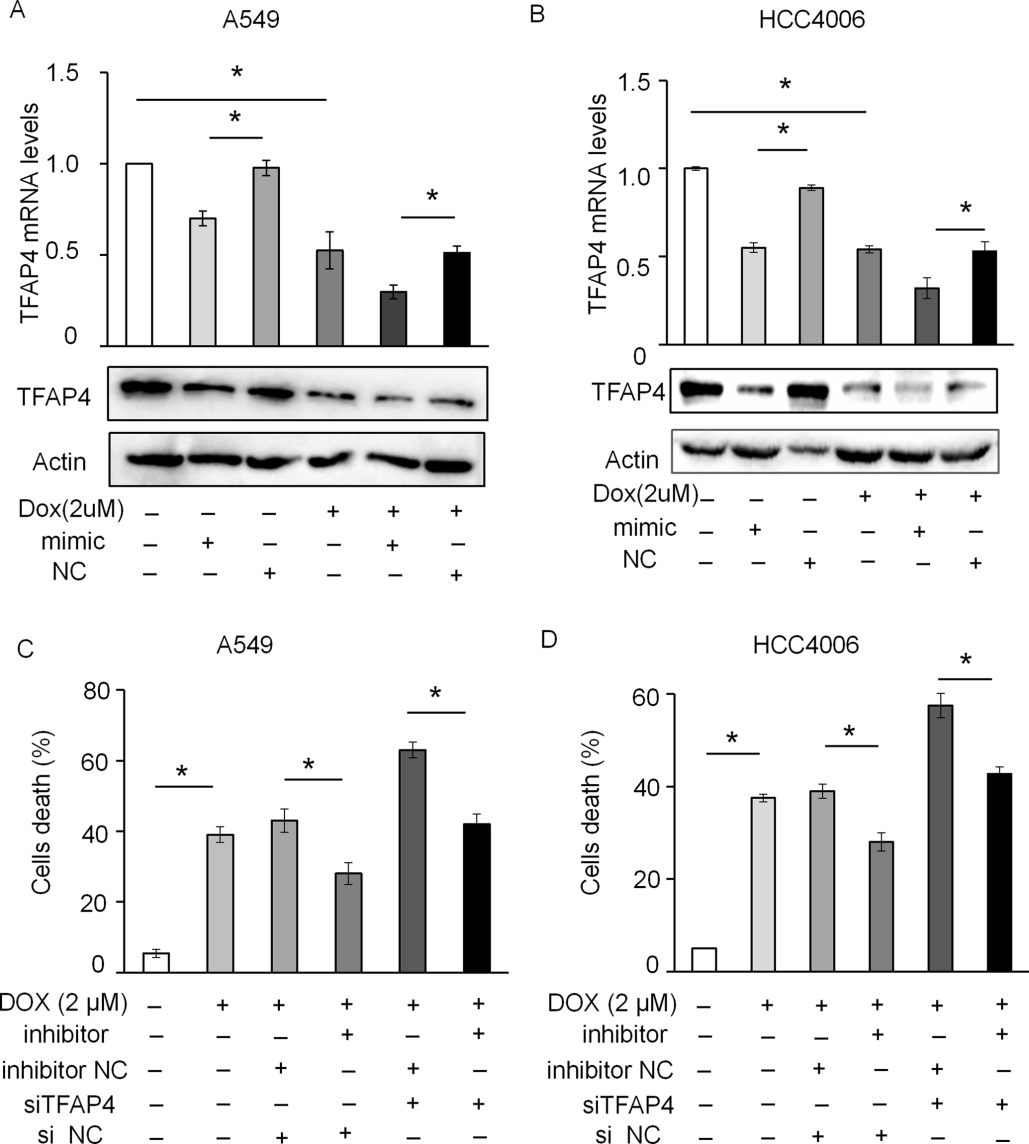
miR-608 regulates DOX-induced apoptosis by targeting TFAP4. The levels of TFAP4 mRNA and protein expression were determined by RT-qPCR and Western blot assay in A549 **(A)** and HCC4006 **(B)** cells treated with 2 μM DOX. Trypan blue staining results showing the effect of TFAP4 silencing on DOX-induced cell death in A549 **(C)** and HCC4006 **(D)** cells transfected with miR-608 inhibitor. Each experiment was independently repeated ≥3 times. The data is presented as the mean ± standard deviation. Student’s t-test, *P < 0.05. miR, miRNA; DOX, doxorubicin; TFAP4, transcription factor activating enhancer-binding protein 4.

siRNA-targeting of TFAP4 markedly inhibited the expression of TFAP4 at the mRNA and protein level in A549 (**Figure 7A**) and HCC4006 cells (**Figure 7B**). Knockdown of TFAP4 alone had no significant effect on cell death (**Supplementary Figures 4A,B and 4B**), while following DOX treatment (2 μM), the expression levels of TFAP4 were downregulated at the mRNA level in A549 (**Figure 7C**) and HCC4006 cells (**Figure 7D**). Furthermore, knockdown of TFAP4 sensitized A549 cells (**Figure 7E**) and HCC4006 cells (**Figure 7F**) to low-dose DOX-induced cell death. We further clarified that in the absence of DOX treatment, the expression of TFAP4 was decreased by miR-608 overexpression, and when exposed to DOX, the levels of TFAP4 decreased overall, particularly in the DOX/miR-608 overexpression co-treatment groups (**Figures 8A, B**). We then treated A549 and HCC4006 cells with a high concentration of DOX (2 μM). The results in **Figures 8C, D** showed that high-dose DOX treatment effectively induced cell death to ∼40%, and that miR-608-knockdown attenuated DOX-induced apoptosis. Furthermore, TFAP4-silencing was able to reverse the decline in cell death caused by the knockdown of miR-608. Collectively, our results demonstrated that following DOX treatment, miR-608 is able to regulate apoptosis in NSCLC cells by targeting TFAP4.

We also examined the effect of TFAP4 on the proliferation and migration of A549 and HCC4006 cells. MTT assay showed that the proliferation was not changed as a result of TFAP4-silencing in A549 cells (**Supplementary Figure 5A**) and HCC4006 cells (**Supplementary Figure 5B**). However, silencing TFAP4 suppressed metastasis, as determined by wound healing (**Supplementary Figure 5C**) and Transwell assays (**Supplementary Figures 5D,E and 5E**).

### TFAP4 Expression Level Is Significantly Increased in NSCLC Tissues

**Figure 9:**
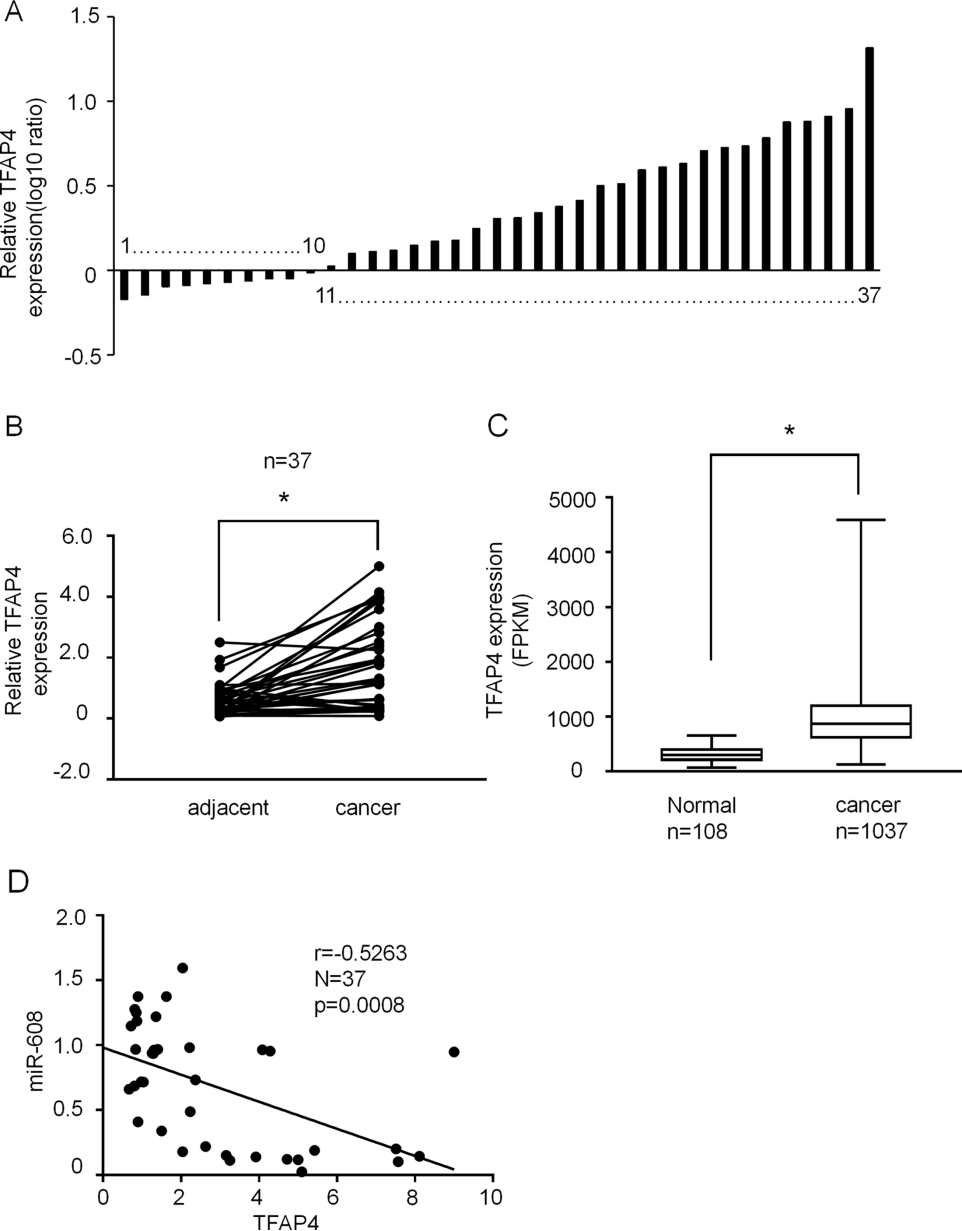
The expression of TFAP4 in NSCLC tissues. **(A)** Result showing the relative expression level of TFAP4 (log10 ratio) in 37 pairs of samples (NSCLC tissues and matched normal tissues) by RT-qPCR. **(B)** Comparison of TFAP4 expression in NSCLC tissues and matched normal tissues analyzed by non-parametric test (n = 37). For comparison, the expression level of TFAP4 in matched normal tissue of NSCLC patient 1 was set to 1. Student’s t-test, *P < 0.05. **(C)** The data from The Cancer Genome Atlas database showing the expression TFAP4 in NSCLC tissues. Student’s t-test, *P < 0.05. (D) The association between miR-608 and TFAP4 expression was analyzed using Spearman’s analysis; R = −0.5263; P < 0.05. TFAP4, transcription factor activating enhancer-binding protein 4; NSCLC, non-small cell lung cancer; miR, miRNA.

The expression levels of TFAP4 in 37 human NSCLC and matched adjacent normal tissues were assessed using RT-qPCR. As shown in **Figure 9A**, TFAP4 expression was upregulated in the NSCLC tissues of 73% (30/37). Non-parametric test showed that TFAP4 expression was significantly increased in NSCLC tissues compared with that in matched adjacent normal tissues (P < 0.05) (**Figure 9B**). Moreover, high expression of TFAP4 was correlated with the clinical index of lymph node metastasis (**Table 2**). Database analysis showed that TFAP4 is significantly correlated with the prognosis of patients (**Supplementary Figure 6A**). Additionally, data from The Cancer Genome Atlas also indicated high expression levels of TFAP4 in NSCLC tissues (**Figure 9C**). The data from **Figure 9D** revealed a negative correlation between the expression levels of miR-608 and TFAP4 in NSCLC tissues, which represents a statistically significant difference.

## Discussion

Accumulating evidence has suggested that miRNAs are frequently dysregulated in a number of diseases and play important regulatory roles in different types of cancer. Because miRNAs can act as both oncogenes and tumor suppressors, they are associated with the initiation and progression of cancer (Fanini and Fabbri, 2016); additionally, miRNA polymorphisms have been linked to the susceptibility of different cancers (Ke et al., 2013; Zheng et al., 2013; Liu et al., 2016). The SNPs in miRNA genes may influence the processing and/or target selection of miRNAs and therefore may be associated with the risk of developing cancer. In this study, we identified that the rs4919510 polymorphism located in the fourth position of miR-608-3p was not associated with susceptibility to NSCLC. We also revealed that rs4919510 did not affect the expression levels of mature miR-608 or its apoptotic function. Although the association between miRNA SNPs and the risk of developing various cancers has been extensively investigated, there are only a few reports addressing the association between these SNPs and the risk of developing NSCLC. Further studies are required to explore the roles of the miR-608 rs4919510 polymorphism in NSCLC.

miR-608 is located in the intron region of the SEMA4G gene on chromosome 10q24.31; miR-608 harbors a SNP (rs4919510) in bp 22 of its mature 25 bp sequence. Currently the association between miR-608 rs4919510 and cancer susceptibility is controversial. Previous studies have illustrated that miR-608 rs4919510 was associated with the incidence of different types of cancer (Huang et al., 2012; Qiu et al., 2015b; Wei et al., 2015; Ying et al., 2016), yet conflicting results suggest that rs4919510 is not associated with cancer susceptibility (Peterlongo et al., 2012; Zhang et al., 2015; Dai et al., 2016; Morales et al., 2016; Ranjbar et al., 2018). Research into miR-608 rs4919510 occurrence and lung cancer susceptibility are equally controversial (Li et al., 2016; Yin et al., 2016). Therefore, further investigation is needed to elucidate this relationship. As in the majority of the previous studies, our study was limited by a relatively small sample population, which may have subsequently influenced the results. In addition, the environment, region, nationality, gender, age, and smoking habits of the participants may have affected the relationship between the miR-608 polymorphism and the incidence of lung cancer.

miRNA SNPs can alter miRNA maturation or change their target sites, ultimately resulting in altered function (Ding et al., 2013). One of the mechanisms influencing susceptibility to tumors might be that polymorphism influences the maturation of miR-608. In our study, we investigated the C to G polymorphism (rs4919510) located at the 37rd position of pre-miR-608. Our data showed that the miR-608-G allele displayed almost the same efficiency to produce mature miR-608 compared with the miR-608-C allele, suggesting that the miR-608 rs4919510 polymorphism did not affect the maturation of miR-608 in A549 and HCC4006 cells, and therefore may not affect the incidence of lung cancer. Another factor to influence changes in tumor susceptibility may be the altered binding capacity of the miRNA to the target gene. miRNA SNPs might confer reduced, or even a total loss of the ability to interact with the 3′ UTR of target mRNAs, hence reducing the capacity of the miRNA for target suppression (Zhao et al., 2013). miRNAs function mainly in the 2–8 base position of the core region; however, miR-608 rs4919510 is located in the fourth position of miR-608-3p, not the core region, so this polymorphism cannot affect its function. The present study confirmed that both miR-608-C and miR-608-G promote apoptosis in A549 and HCC4006 cells, thus demonstrating that the rs4919510 polymorphism affects neither the maturation of miR-608 nor its ability to bind to the target gene, and ultimately does not affect the function of miR-608. However, the exact mechanism of SNPs in disease occurrence remains unclear. Therefore, more research should be conducted to clarify the relationship between miRNA SNPs and their influencing mechanisms in the occurrence of disease

Previous studies have indicated that miR-608 is downregulated in malignant tumors and acts as a tumor suppressor in a number of cancers, including hepatocellular carcinoma, glioma, colon cancer, and bladder cancer (Wang et al., 2016a; Wang et al., 2016b; Yang et al., 2016; Liang et al., 2017). In our present work, we observed similar results in NSCLC samples, indicating that miR-608 may act as tumor suppressor gene in the development and progression NSCLC.

TFAP4 is an important, recently discovered transcriptional regulator that belongs to the basic helix-loop-helix-leucine zipper (bHLH-LZ) transcription factor family (Jackstadt et al., 2013). TFAP4 plays a carcinogenic role by promoting cell proliferation, invasion, and metastasis, and by suppressing the expression of cell cycle arrest-associated genes and apoptosis (Jackstadt et al., 2013; Song et al., 2018). Although TFAP4 has been studied in other cancers, the oncogenic mechanism and function of TFAP4 in lung cancer are not clearly understood. In our study, we found that TFAP4 expression levels were increased in NSCLC tissues, and that this resulted in the inhibition of DOX-induced apoptosis. We also discovered that TFAP4 silencing was able to suppress cell migration. These results provide new insights into the role of TFAP4 in NSCLC, though the clinical significance and specific mechanisms of TFAP4 and miR-608 in lung cancer progression require further investigation.

## Conclusions

In summary, our study revealed that in the Han Chinese population of Qingdao city and the surrounding regions, miR-608 was downregulated, while TFAP4 was upregulated in the tumor tissues of patients with NSCLC. Additionally, the miR-608 rs4919510 polymorphism was not associated with susceptibility to NSCLC. *In vitro*, miR-608 appeared to promote DOX-induced apoptosis by negatively regulating TFAP4 expression in NSCLC tissues. These findings may assist in the construction of effective treatment regimens for patients with NSCLC.

## Data Availability

All datasets generated for this study are included in the manuscript and the supplementary files.

## Ethics Statement

This study was carried out in accordance with the recommendations of Clinical Research Specifications and Guidelines hospital-based ethics committee of Affiliated Hospital of Qingdao University with written informed consent from all subjects. All subjects gave written informed consent in accordance with the Declaration of Helsinki. The protocol was approved by the hospital-based ethics committee of Affiliated Hospital of Qingdao University.

## Author Contributions

J-XW and HG designed the project, while Y-FW, DD, W-XZ, and S-HD performed the associated experiments. The data were analyzed by XA, HG, YL, W-JJ, Y-QH, and ZY, who also donated materials and analysis tools. Y-FW, XA, and J-XW contributed to the writing of the manuscript. All authors have read and approved the final manuscript.

## Funding

The present study was supported by the National Natural Science Foundation of China (grant no. 81622005 to JW, no. 81802822 to XA) and the Natural Science Foundation of Shandong Province (grant no. JQ201815 to JW, no. ZR2018BH017 to XA).

## Conflict of Interest Statement

The authors declare that the research was conducted in the absence of any commercial or financial relationships that could be construed as a potential conflict of interest.
