# Corrigendum: MicroRNA-608 Promotes Apoptosis in Non-Small Cell Lung Cancer Cells Treated With Doxorubicin Through the Inhibition of TFAP4

**DOI:** 10.3389/fgene.2021.649586

**Published:** 2021-03-30

**Authors:** Yi-Fei Wang, Xiang Ao, Ying Liu, Dan Ding, Wen-Jie Jiao, Zhuang Yu, Wen-Xin Zhai, Sheng-Hua Dong, Yu-Qi He, Hang Guo, Jian-Xun Wang

**Affiliations:** ^1^School of Basic Medical Sciences, Qingdao University, Qingdao, China; ^2^Institute for Translational Medicine, Qingdao University, Qingdao, China; ^3^Affiliated Hospital, Qingdao University, Qingdao, China; ^4^Department of Gastroenterology, The Seventh Medical Center of PLA General Hospital, Beijing, China; ^5^Department of Anesthesiology, The Seventh Medical Center of PLA General Hospital, Beijing, China

**Keywords:** microRNA-608, single nucleotide polymorphisms, apoptosis, transcription factor activating enhancer-binding protein 4, non-small cell lung cancer

In the original article, there were two mistakes in Figure 5 as published. (1) In Figure 5C, the scale bar didn't match the image. (2) We inadvertently used the apparent duplication in different groups (Con, NC, and Mimic) of Figures 5D and 5E during the figure preparation. We found that the errors were caused by our carelessness in exporting the representative images and compiling these figures. The corrected Figure 5 appears below.

**Figure 5:**
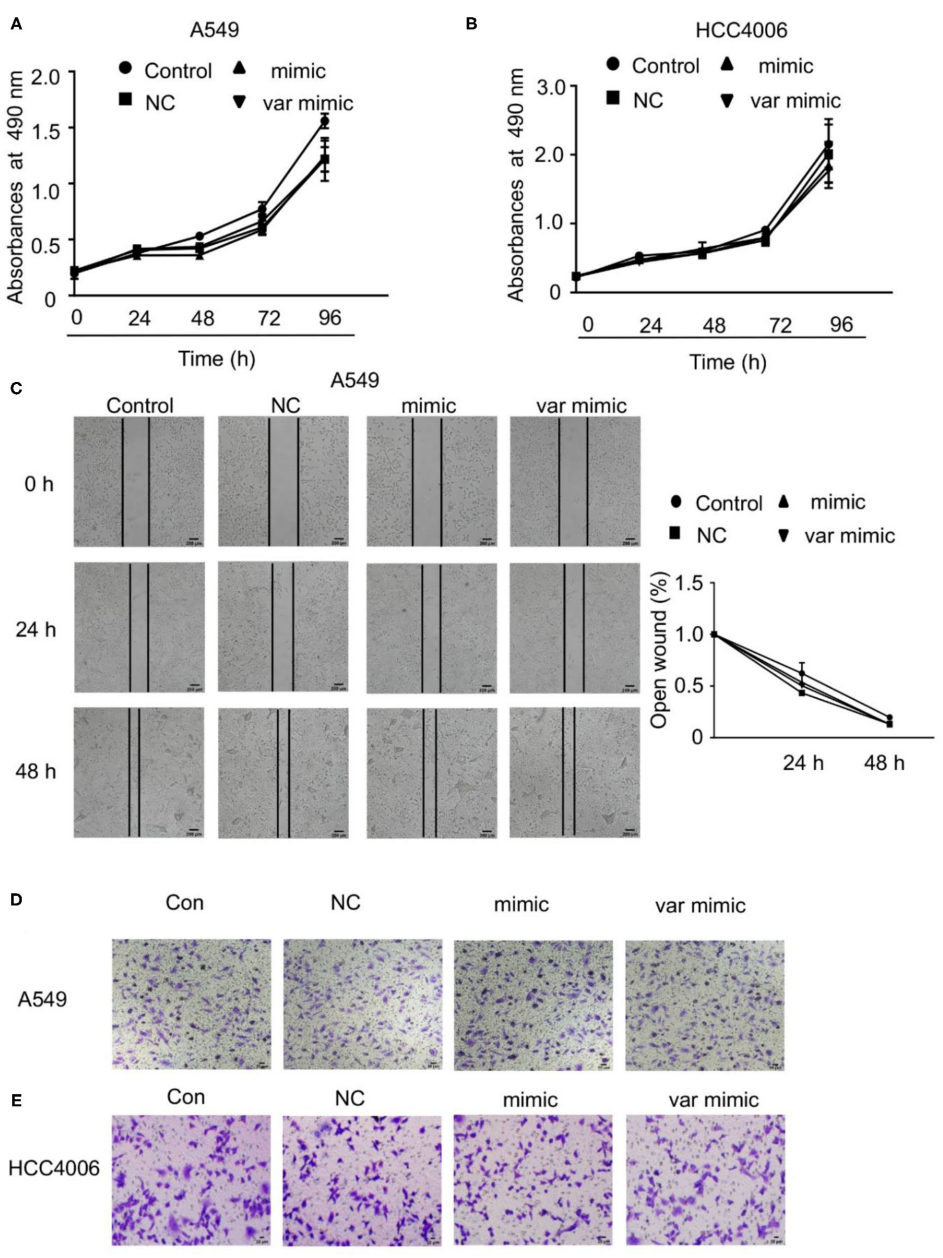
Effect of miR-608 on the proliferation and metastasis of NSCLC cells. Cell proliferation was examined by MTT assay in A549 **(A)** and HCC4006 **(B)** cells transfected with miR-608 mimic or var mimic for the indicated times. **(C)** The effect of miR-608 on metastasis determined by wound-healing assay in A549 cells. Transwell assay results showing the effect of miR-608 on metastasis in A549 cells **(D)** and HCC4006 **(E)** cells. miR, microRNA.

The authors apologize for this error and state that this does not change the scientific conclusions of the article in any way. The original article has been updated.

