# Supplementary figures and images for: MicroRNA-608 Promotes Apoptosis in Non-Small Cell Lung Cancer Cells Treated With Doxorubicin Through the Inhibition of TFAP4
Source: Front Genet. 2019 Sep 10;10:809. doi: 10.3389/fgene.2019.00809 (PMC6746977; doi:10.3389/fgene.2019.00809)

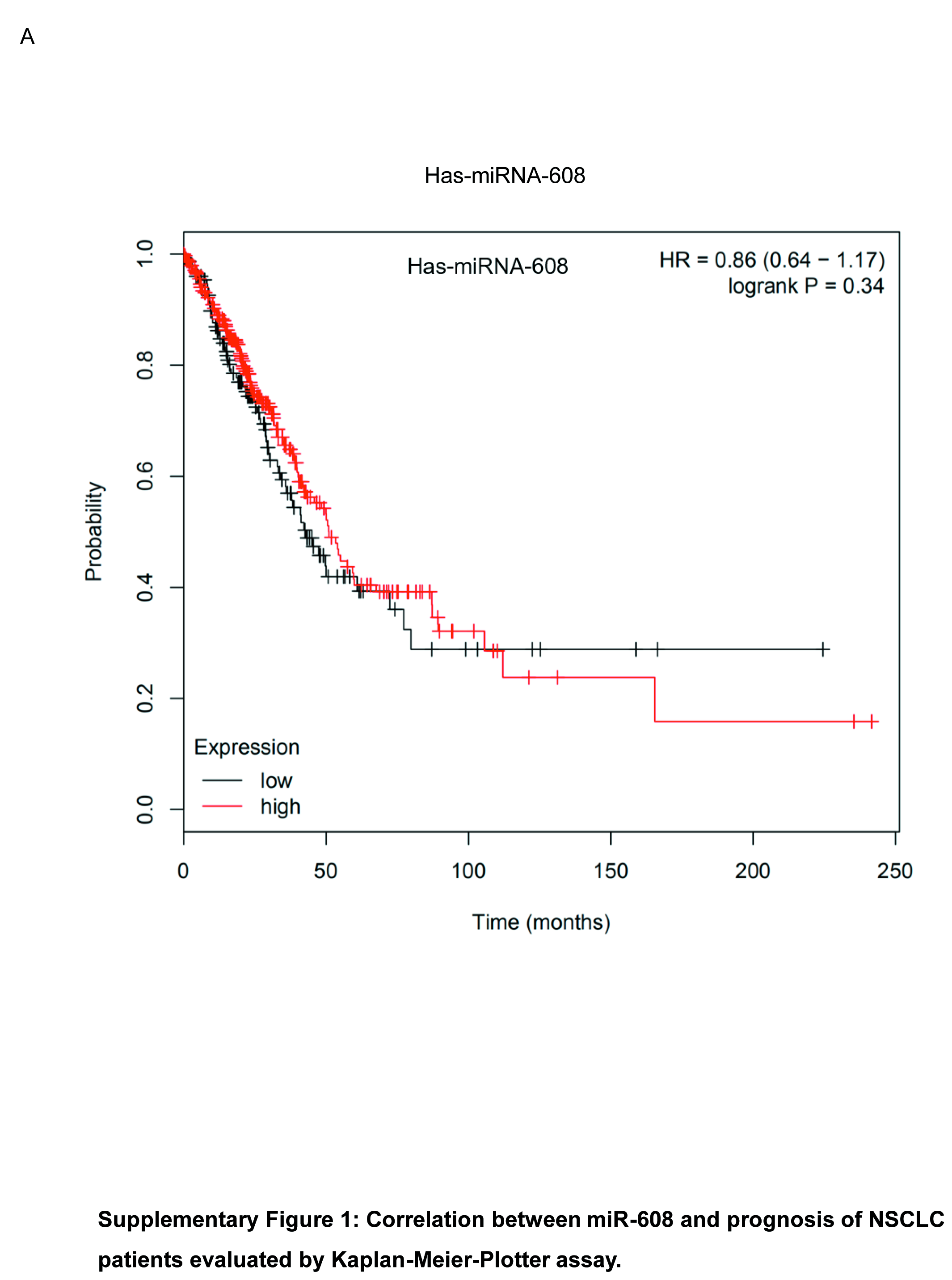

Supplement: Supplementary file 1 [file Image_1.tif]

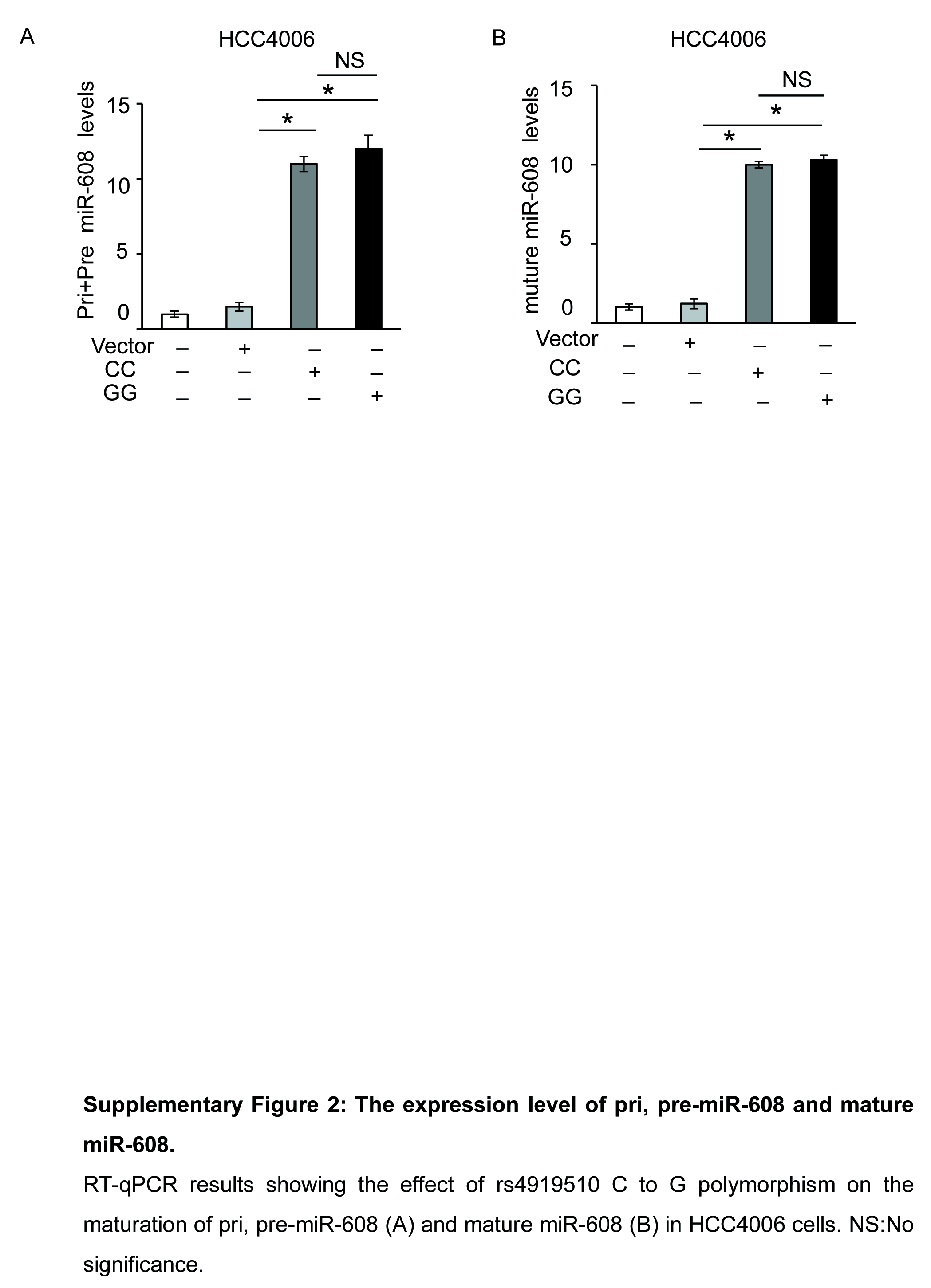

Supplement: Supplementary file 2 [file Image_2.tif]

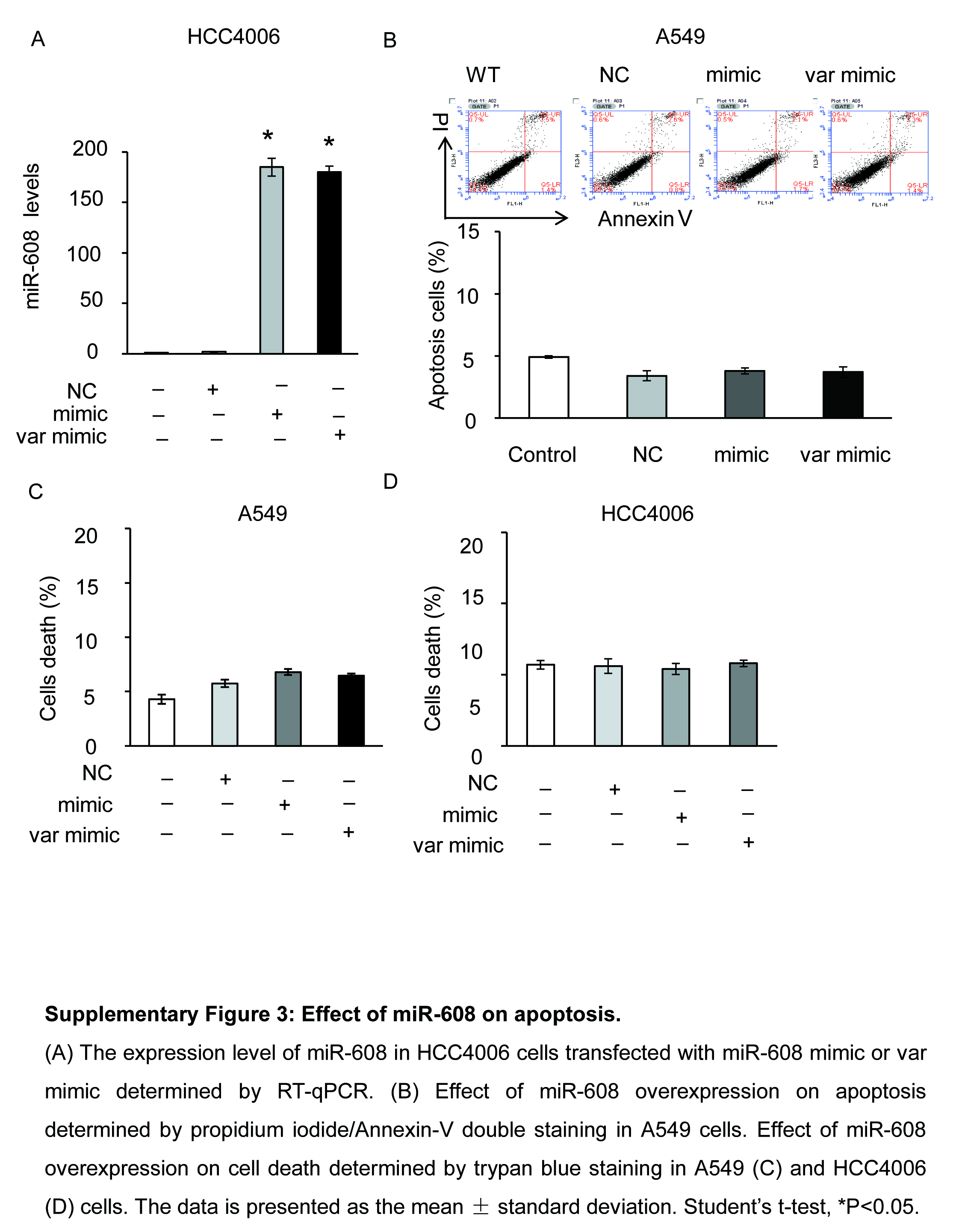

Supplement: Supplementary file 3 [file Image_3.tif]

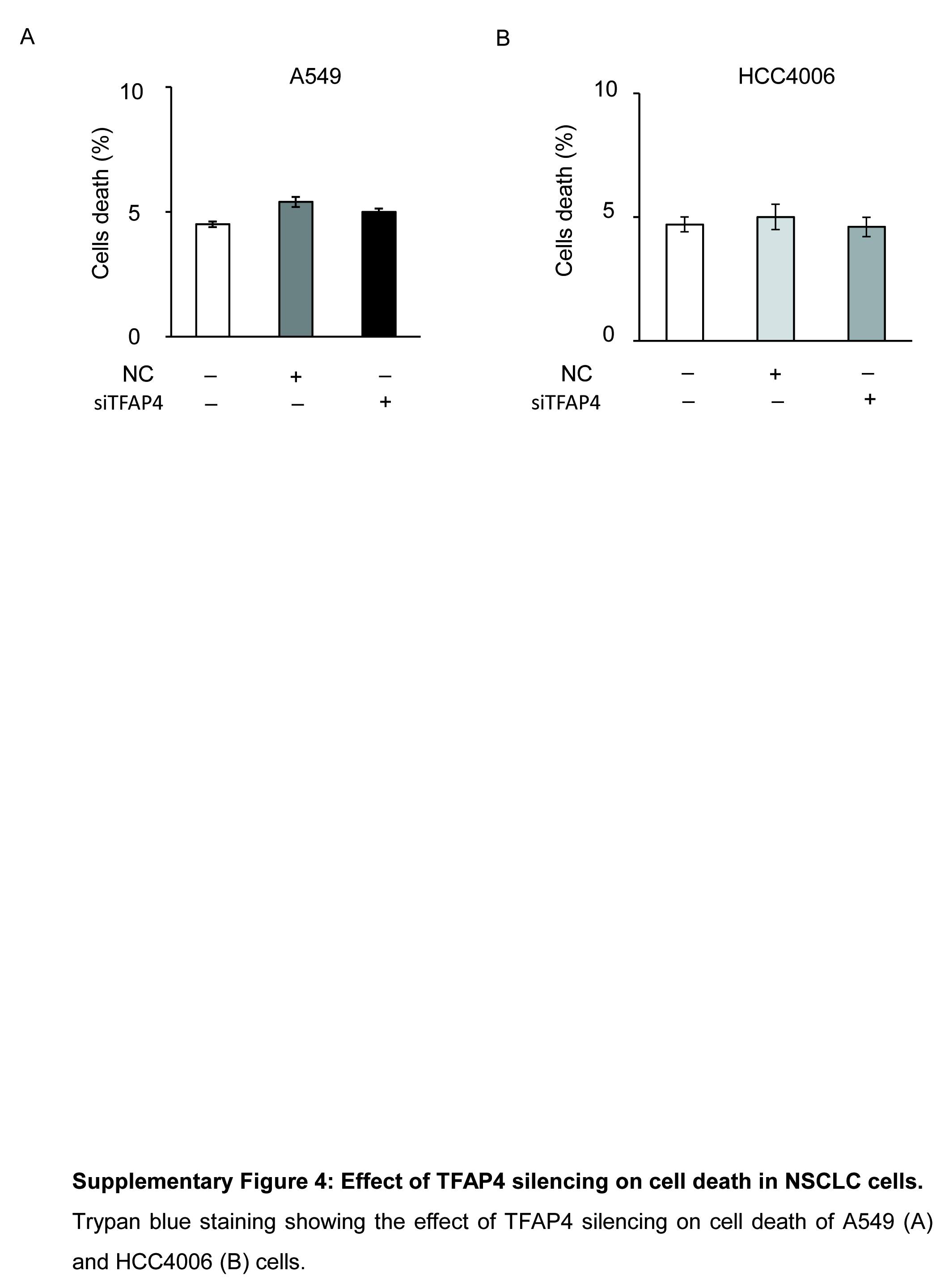

Supplement: Supplementary file 4 [file Image_4.tif]

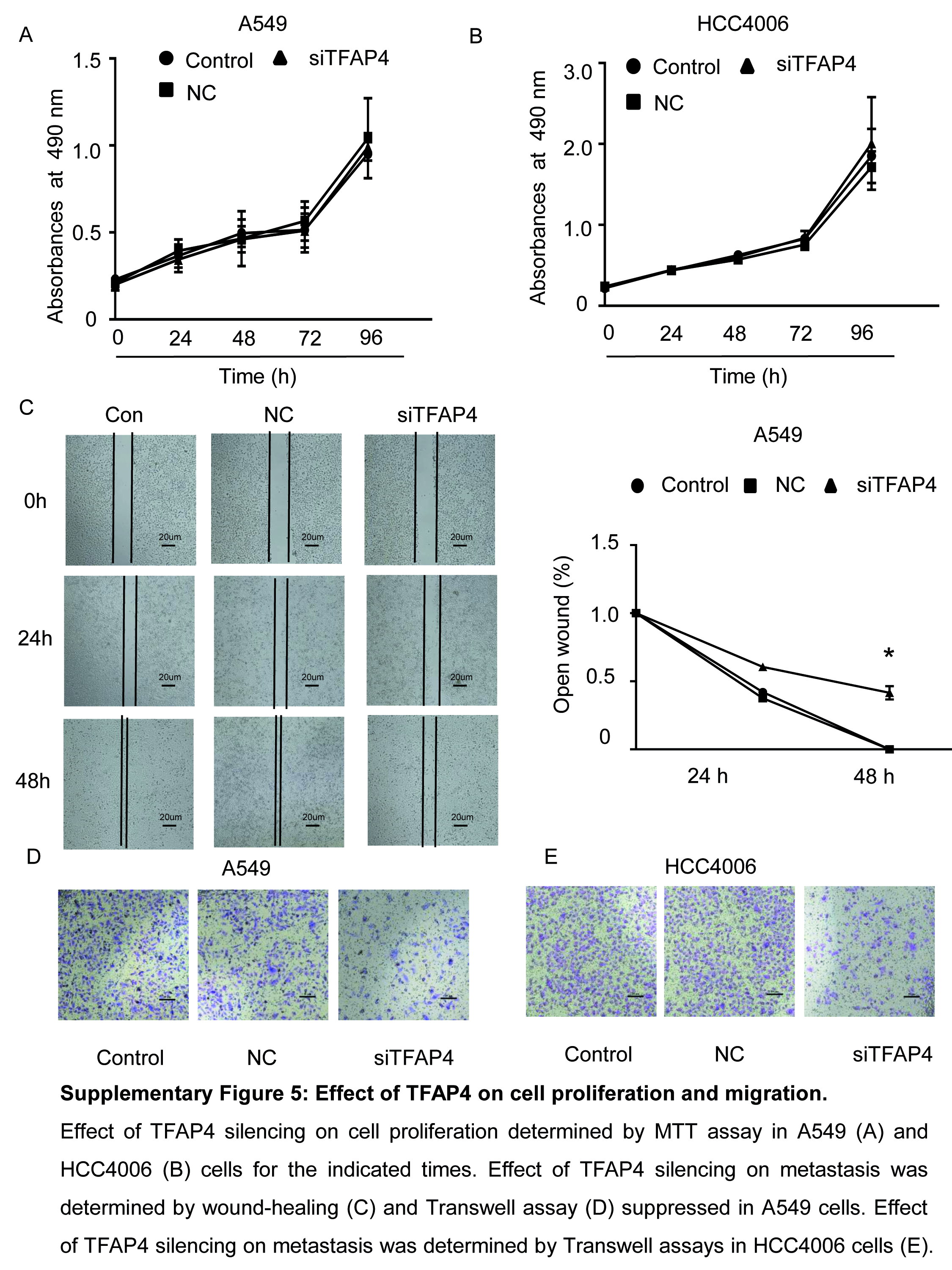

Supplement: Supplementary file 5 [file Image_5.tif]

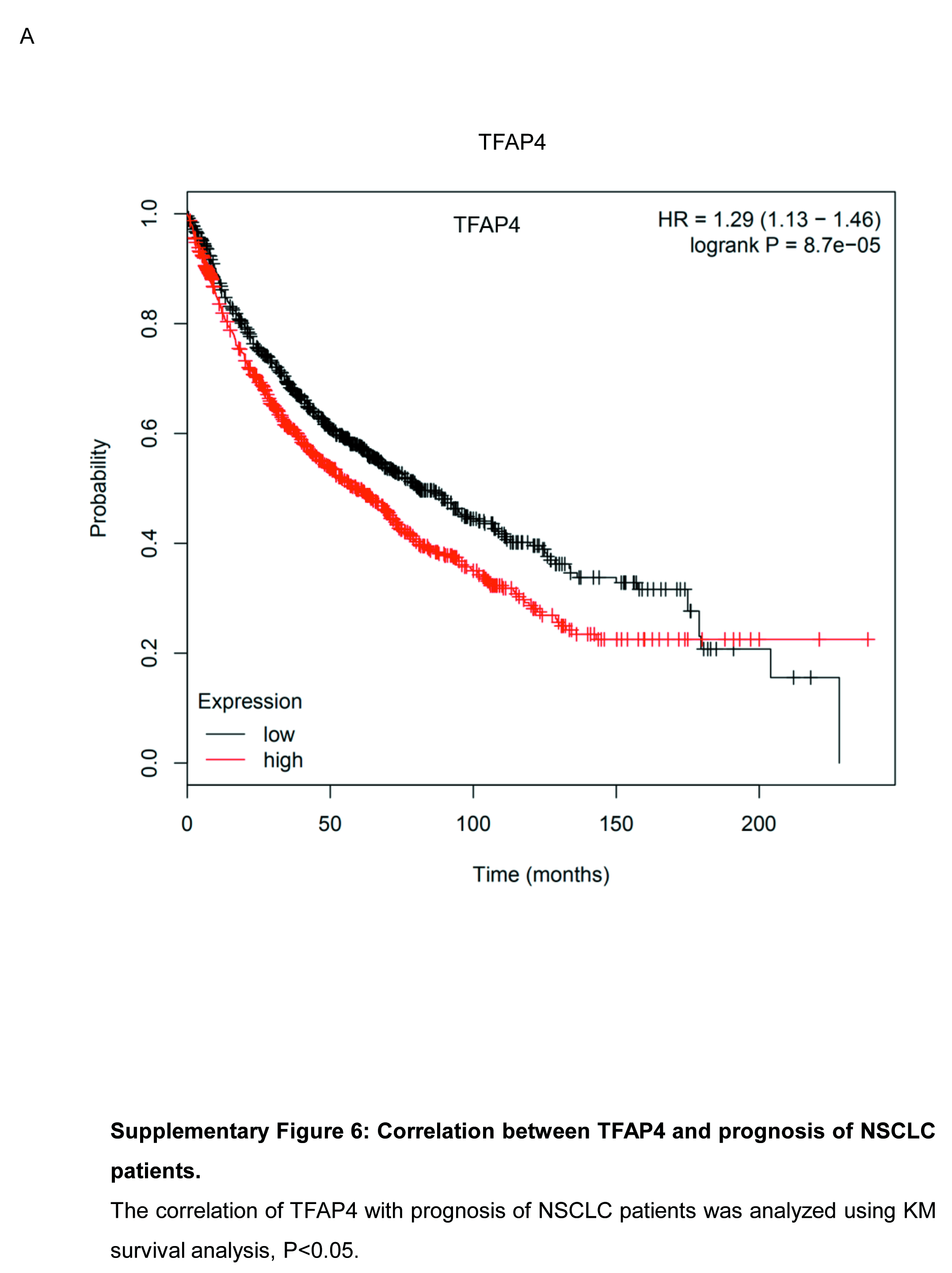

Supplement: Supplementary file 6 [file Image_6.tif]
