# Supplementary material for: MicroRNA-608 Promotes Apoptosis in Non-Small Cell Lung Cancer Cells Treated With Doxorubicin Through the Inhibition of TFAP4
Source: Front Genet. 2019 Sep 10;10:809. doi: 10.3389/fgene.2019.00809 (PMC6746977; doi:10.3389/fgene.2019.00809)
Supplement: Supplementary file 7 [file Table_1.docx]

**Supplementary Table 1. Characteristics of patients with Non-small cell lung cancer**

| **Clinicopathologic features** | **n** |
| --- | --- |
| **Age (years)** |  |
| >55 | 28 |
| ≤55 | 9 |
| **Gender** |  |
| Female | 16 |
| Male | 21 |
| **Tumor size (cm)** |  |
| <4 | 35 |
| ≥4 | 2 |
| **Tumor invasion depth (T)** |  |
| T1 | 32 |
| T2 | 5 |
| T3 | 0 |
| T4 | 0 |
| **Lymph node metastasis (N)** |  |
| N0 | 34 |
| N1 | 3 |
| N2 | 0 |
| N3 or above | 0 |
| **Distant metastasis (M)** |  |
| M0 | 35 |
| M1 | 2 |
| **TNM stage** |  |
| Ⅰ-Ⅱ | 35 |
| Ⅲ-Ⅳ | 2 |
| **Type of diagnosis** |  |
| adenocarcinoma | 31 |
| squamous cell carcinoma | 5 |
| large cell carcinoma | 1 |
