# Supplementary material for: MicroRNA-608 Promotes Apoptosis in Non-Small Cell Lung Cancer Cells Treated With Doxorubicin Through the Inhibition of TFAP4
Source: Front Genet. 2019 Sep 10;10:809. doi: 10.3389/fgene.2019.00809 (PMC6746977; doi:10.3389/fgene.2019.00809)
Supplement: Supplementary file 8 [file Table_2.pdf]

Supplementary Table 2. Sequences of primers used in this experiment

Primers for qPCR	sequence(5'---3')
miR-608-F	GGTGTTGGGACAGCTCCGTA
miR-608-R	GTATCAACGCAGAGTACTTT
Pre miR-608-F	CCAGGGGTGGTGTTGGGA
Pre miR-608-R	AAGAGGCAGCCTTTGATGGA
TFAP4-F	GTGCCCACTCAGAAGGTGC
TFAP4-R	GGCTACAGAGCCCTCCTATCA
TAp63-F	ATGTCCCAGAGCACACAG
TAp63-R	AGCTCATGGTTGGGGCAC
△Np63-F	CAGACTCAATTTAGTGAG
△Np63-R	AGCTCATGGTTGGGGCAC
TFE3-F	CCCCTGCCATGTCGTCATC
TFE3-R	GTGGACGGCTCAATGTGTG
TGFβ1-F	GGCCAGATCCTGTCCAAGC
TGFβ1-R	GTGGGTTTCCACCATTAGCAC
MAPK13-F	GAGAAGGTGGCCATCAAGAA
MAPK13-R	GTCCTCATTCACAGCCAGGT
PIK3CA-F	TGCTAAAGAGGAACACTGTCCA
PIK3CA-R	GGTACTGGCCAA AGATTCAAAG
human U6-F	CTCGCTTCGGCAGCACA
human U6-R	AACGCTTCACGAATTTGCGT
human GAPDH-F	GGACTCATGACCACAGTCCATGCC
human GAPDH-R	TCAGGGATGACCTTGCCCACAG
**Primers for genotyping**	**sequence(5'---3')**
miR-608-F	TCTGGCTAGGTAATGGCTCC
miR-608-R	GCATCTGTGGCCTTCCATGA
**Primers for plasmid construction**	**sequence(5'---3')**
MiR-608-F	TCTGGCTAGGTAATGGCTCC
MiR-608-R	CCCAGACCTACTAAGTTTAGCATCT
TFAP4-WT-F	CTGTAAATCGACCTTTGAACGAAG
TFAP4-WT-R	GAGAGAGACAGACATTTTTGGGG
TFAP4-Mut1-F	ATTCGCCACTCTCAACACCCCCAGCCCCCACCTCT
TFAP4-Mut1-R	GTGTTGAGAGTGGCGAATTCTAGTGCCGAGATG
TFAP4-Mut2-F	CTTAACACCCAAAAATGTCTGTCTCTCTCCCATG
TFAP4-Mut2-R	GACATTTTTGGGTGTTAAGATGAGACCTGGAGGCAGAGG
